# Tailoring the composition of novel wax esters in the seeds of transgenic *Camelina sativa* through systematic metabolic engineering

**DOI:** 10.1111/pbi.12679

**Published:** 2017-02-02

**Authors:** Noemi Ruiz‐Lopez, Richard Broughton, Sarah Usher, Joaquin J. Salas, Richard P. Haslam, Johnathan A. Napier, Frédéric Beaudoin

**Affiliations:** ^1^ IHSM‐UMA‐CSIC Universidad de Málaga Málaga Spain; ^2^ Department of Biological Chemistry Rothamsted Research Harpenden Herts UK; ^3^ Instituto de la Grasa Universitario Pablo de Olavide Seville Spain

**Keywords:** metabolic engineering, seed lipids, Camelina, wax esters

## Abstract

The functional characterization of wax biosynthetic enzymes in transgenic plants has opened the possibility of producing tailored wax esters (WEs) in the seeds of a suitable host crop. In this study, in addition to systematically evaluating a panel of WE biosynthetic activities, we have also modulated the acyl‐CoA substrate pool, through the co‐expression of acyl‐ACP thioesterases, to direct the accumulation of medium‐chain fatty acids. Using this combinatorial approach, we determined the additive contribution of both the varied acyl‐CoA pool and biosynthetic enzyme substrate specificity to the accumulation of non‐native WEs in the seeds of transgenic Camelina plants. A total of fourteen constructs were prepared containing selected FAR and WS genes in combination with an acyl‐ACP thioesterase. All enzyme combinations led to the successful production of wax esters, of differing compositions. The impact of acyl‐CoA thioesterase expression on wax ester accumulation varied depending on the substrate specificity of the WS. Hence, co‐expression of acyl‐ACP thioesterases with *Marinobacter hydrocarbonoclasticus* WS and *Marinobacter aquaeolei* FAR resulted in the production of WEs with reduced chain lengths, whereas the co‐expression of the same acyl‐ACP thioesterases in combination with *Mus musculus* WS and *M. aquaeolei* FAR had little impact on the overall final wax composition. This was despite substantial remodelling of the acyl‐CoA pool, suggesting that these substrates were not efficiently incorporated into WEs. These results indicate that modification of the substrate pool requires careful selection of the WS and FAR activities for the successful high accumulation of these novel wax ester species in Camelina seeds.

## Introduction

There is a growing recognition of the potential of so‐called green factories to help address some of the major societal challenges that now face the human race (Yuan and Grotewold, 2015). In particular, the ultimate need (irrespective of timescale) to transition from dependence on fossil fuels such as petroleum to more renewable and sustainable sources remains a very high priority (Beaudoin *et al*., 2014; Carlsson *et al*., 2011; Vanhercke *et al*., 2013). Although it is now generally accepted, if only because of the volumes required, that bio‐renewable forms could not hope to replace the current utilization levels of fossil fuels for combustible energy, it is equally appreciated that bio‐based compounds could substitute for other, less voluminous petrochemically derived forms (Carlsson *et al*., 2011). These could include acting as replacements for the chemical feedstocks used to create plastics and polymers such as nylon, and also to substitute for fossil fuel‐derived lubricants (Bates *et al*., 2013). In particular, a renewable, bio‐based lubricant would be highly desirable, combining sustainability with improved biodegradability (Carlsson *et al*., 2011). However, the demanding physicochemical properties which define a high‐performance lubricant such as might be found in vehicle transmission or hydraulic systems preclude the use of most currently available forms of biologically derived compounds (Jaworski and Cahoon, 2003). For example, oils (usually in the form of triacylglycerols—TAGs) derived from plant seeds lack both the thermal stability and melting point normally associated with petrochemically derived compounds. However, one naturally occurring compound which has been proven to have utility as a biolubricant is spermaceti oil, used extensively in the first half of the 20th century in the automotive industry (Iven *et al*., 2016). Remarkably, it was even used in the NASA Apollo ‘moon‐shots’ of the 1960s, reportedly on the basis of its superior ability to withstand extremes of temperature and pressure (Hoare, 2009). However, as sourcing this compound required the killing one of the largest sentient mammals on the planet, good sense prevailed and the commercial hunting of whales for the oil has been globally banned since the 1980s.

Yet interest has remained in understanding better the particular properties of spermaceti oil and the identification of alternative, less destructive, sources. In fact, spermaceti oil is predominately comprised of wax esters (WE, 76%) and significantly lower amounts of TAGs (23%), and it is the presence of the WE (and their associated ester‐bonded acyl‐alcohols) that confers its desirable properties (Nevenzel, 1970). Unfortunately, although higher plants make a wide variety of different oils and fatty acids (Napier, 2007), there are hardly any known examples of plant species which accumulate WE in their seeds, the best characterized example being jojoba (*Simmondsia chinensis*) (Dyer *et al*., 2008). Jojoba is a desert shrub which is poorly adapted for modern agriculture, and the specific form of WE present in the plant is distinct in its chemical properties to the WEs found in spermaceti oil (Miwa, 1971). Thus, in terms of both composition and production capacity, jojoba is not a viable substitute for spermaceti oil. However, the biosynthesis of jojoba WEs has provided insights into how these useful compounds are made, and the identification of the key enzymes and genes has permitted a biotechnological approach, in which the capacity to synthesize WEs is transferred to a suitable agronomically viable host crop (Lardizabal *et al*., 2000; Metz *et al*., 2000).

**Figure 1:**
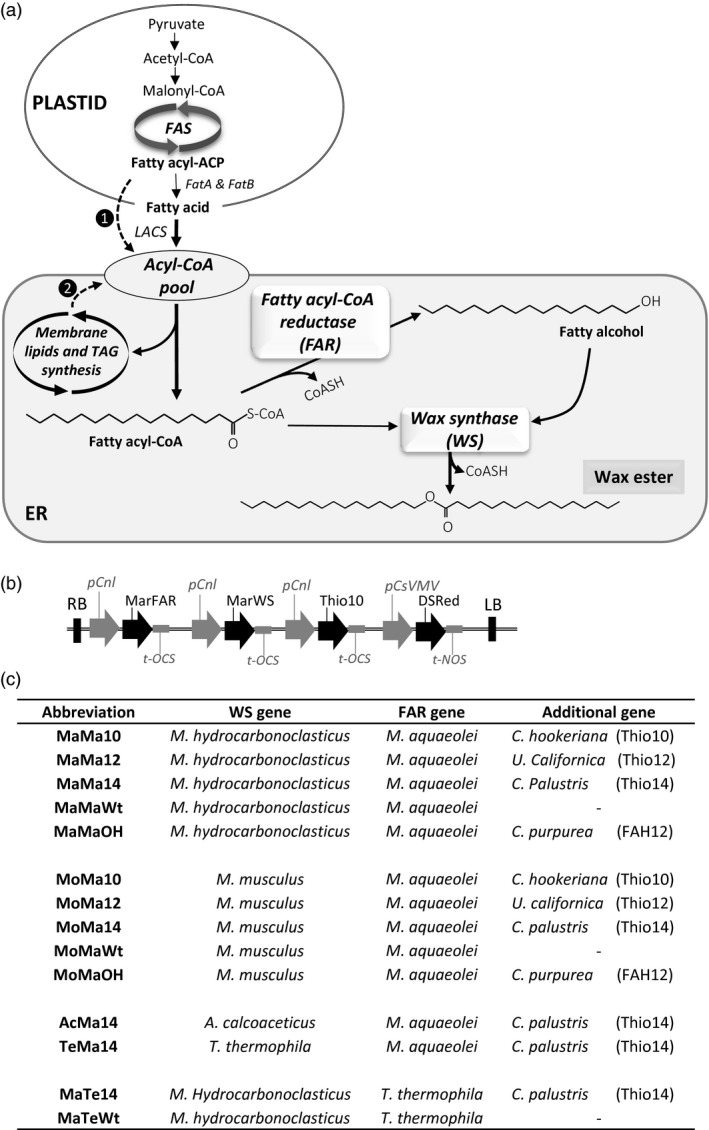
Metabolic engineering strategy for tailoring the composition of Wax Ester (WE) produced in transgenic Camelina seeds. (a) Schematic illustration of the approach used for investigating the contribution of heterologous enzymes activity and the acyl‐CoA substrate pool to wax ester content and composition. ❶ Modulation of the acyl‐CoA substrate pool by the co‐expressing acyl‐ACP thioesterases which prematurely terminate fatty acid synthesis in the plastid resulting in the accumulation of medium‐chain fatty acids. ❷ Acyl exchange pathway(s) required for the transfer of unusual fatty acids (e.g. hydroxylated fatty acids) from membrane glycerolipids to the acyl‐CoA substrate pool. The enzymes required for WE synthesis in the endoplasmic reticulum (ER) are a fatty acyl‐CoA reductase (FAR) and a wax synthase (WS). (b) Diagram of the constructs used for seed‐specific expression of different combinations of wax biosynthetic and lipid/fatty acid modifying enzymes. (c) Combinations of wax biosynthetic and lipid modifying genes tested for manipulating the composition of WEs produced in Camelina seeds. A simple abbreviated nomenclature was adopted for the definition of each gene set. The first two letters of each gene combination represent the wax synthase, the next two letters the fatty alcohol reductase and the last two the presence or the absence (Wt) of an additional gene. For example, the abbreviation MoMa10 indicates the combination of Mouse WS, *Ma* *rinobacter* FAR and *Cuphea hookeriana* acyl‐ACP C10 thioesterase (Thio10). MoMaWt represents the same WS and FAR combination in a wild‐type fatty acid background.

Based on studies in jojoba and other WE‐accumulating organisms (such as some bacteria and protists), it is known that synthesis of WEs proceeds via a two‐step reaction—firstly the conversion of an acyl chain to a fatty alcohol via a fatty acid reductase (FAR), followed by the condensation of the fatty alcohol with a fatty acid by a wax synthase (WS) to yield the final product, the WE (Figure 1a; Ishige *et al*., 2003; Jaworski and Cahoon, 2003). In both cases, the enzymes utilize acyl‐CoAs as their substrates and the reactions are predicted to occur in the endoplasmic reticulum. Polypeptides associated with the FAR and WS activities were purified from jojoba microsomal membrane fractions, knowledge which allowed the further identification of cDNA clones encoding these enzymes (Lardizabal *et al*., 2000; Metz *et al*., 2000). Functional characterization in transgenic plants demonstrated the predicted activities, opening the possibility of producing ‘tailored wax esters’ in the seeds of a suitable host plant (Lardizabal *et al*., 2000; Metz *et al*., 2000). However, despite these early successes, there has been only limited progress in increasing and optimizing the accumulation of WEs in seeds, and it is equally clear that the host plant plays an important role in determining the levels achieved (Dyer *et al*., 2008; Iven *et al*., 2016; Zhu *et al*., 2016; Horn *et al*., 2013). Moreover, a significant challenge remains in terms of directing the flux of substrate acyl‐CoAs towards FAR and WS whilst minimizing their incorporation into TAG (which represents a metabolic dead end as far as WE synthesis is concerned). This must be balanced with a requirement to synthesize sufficient TAG to allow seed germination (Bates *et al*., 2013), and also to avoid the overaccumulation of deleterious fatty alcohols which might arise from nonstoichiometric activities of FAR and WS. Finally, the composition of the fatty acids in the acyl‐CoA pool may not be well matched with the substrate preferences of the FAR and WS enzymes, resulting in low overall synthesis of WEs. Through the adoption of iterative metabolic engineering approaches (Haslam *et al*., 2016), incremental but important gains in our capacity to direct the accumulation of WEs in the seeds of GM plants have been achieved, although these have mainly been focused on producing a jojoba‐like WE in which the majority of acyl chains are longer than 20 carbons (C40 + WEs; Zhu *et al*., 2016). Attempts to generate shorter WEs, which would have physiochemical properties closer to a potential biolubricant, have been primarily focused on evaluating enzyme activities from different source organisms and enhancing substrate flux through cotargeting of the FAR and WS to specific organelles (Iven *et al*., 2013, 2016). In this study, in addition to systematically evaluating a wide panel of WE biosynthetic activities, we have also modulated the acyl‐CoA substrate pool through the co‐expression of acyl‐ACP thioesterases which prematurely terminate plastidial fatty acid synthesis and result in the accumulation of medium‐chain fatty acids and their acyl‐CoA derivatives (Figure 1c; Dehesh *et al*., 1996a,b). By this approach, we determined the combinatorial contribution of both the acyl‐CoA pool and FAR/WS substrate specificity to the accumulation of non‐native WEs in the seeds of transgenic Camelina plants.

## Results and discussion

Camelina was chosen as an experimental platform for seed‐specific engineering as it is an oilseed crop that is amenable to rapid gene testing: it is easily transformed using *Agrobacterium*‐based methods and seed‐specific selection markers (e.g. DsRed) and it has short life cycle (Haslam *et al*., 2016). The general strategy for producing wax esters in seeds of Camelina is shown in Figure 1a. Acyl‐ACP products, primarily 16:0‐and 18:1‐ACP, synthesized by the plastidial fatty acid synthase (FAS) are hydrolysed by the FAtB and FATA thioesterases, respectively, and activated to CoA esters by long‐chain acyl‐CoA synthetases (LACS) during their export from the plastid. At that point, they enter the endoplasmic reticulum (ER)‐associated acyl‐CoA pool where they become available as the primary substrates for wax ester synthesis. As outlined above, this can be achieved by introduction and seed‐specific expression of a fatty acyl reductase (FAR) and a wax synthase (WS) leading the production of novel wax esters. In this study, FAR genes from the bacteria *Marinobacter aquaeolei* (Iven *et al*., 2013) and *Tetrahymena thermophile* (Dittrich‐Domergue *et al*., 2014) were expressed in combinations with WS genes from *Marinobacter hydrocarbonoclasticus*, Mouse (*Mus musculus*) (Cheng and Russell, 2004), *Acinetobacter calcoaceticus* (Kalscheuer *et al*. 2003) and *T. thermophile* (Biester *et al*., 2012). These FAR and WS combinations were also co‐expressed with a number of additional genes (i.e. acyl‐ACP thioesterases or a fatty acid hydroxylase) intended to further modify seed fatty acid composition (Figure 1c). For ease of reference, a simple abbreviated nomenclature was adopted for the definition of each gene set—for example, the abbreviation MoMa10 indicates the combination of Mouse WS, *Ma* *rinobacter* FAR *and Cuphea hookeriana 10:0*‐ACP thioesterase (Thio10).

### Modulation of the substrate pool for wax ester biosynthesis

Earlier studies (Zhu *et al*., 2016) have indicated how the endogenous seed oil fatty acid composition influences the outcome of any metabolic strategy for the production of industrial oils. Furthermore, it can be assumed that the availability of specific acyl‐CoA substrates has a major effect on the composition of the wax ester molecular species produced. Typically, *de novo* fatty acid synthesis in plants generates C16 and C18 fatty acids; however, previous work has shown that the expression of functionally divergent FatB acyl‐ACP thioesterases from Cuphea species in Camelina seeds results in the accumulation of medium‐chain fatty acids with chain lengths ranging from C8 to C14 (Kim *et al*., 2015). To explore how the availability of specific acyl‐CoA substrate might impact wax ester synthesis and composition (Figure 1a; pathway 1), selected acyl‐ACP thioesterases were evaluated using a combinatorial approach. Candidate FatB acyl‐ACP thioesterases were selected from published reports of their substrate specificities: a 10:0‐ACP thioesterases from *Cuphea hookeriana* (Thio 10; Dehesh *et al*., 1996a), a 12:0‐ACP thioesterase from *Umbellularia californica* (Thio12; Voelker *et al*., 1992) and a 14:0‐ACP thioesterase gene from *Cuphea palustris* (Thio14; Dehesh *et al*., 1996b). Analysis of the acyl‐CoA pool in developing seeds of the transgenic Camelina (Table S1) confirmed the suitability of this strategy for manipulating the substrate pool. The cumulative amount of 10:0‐CoA, 12:0‐CoA and 14:0‐CoA was <0.5% in Wt but up to 19.7% of total acyl‐CoAs in MoMa and MaMa lines. Additionally, in an attempt to engineer the accumulation of more unusual (and industrially valuable) acyl moieties in wax esters, the seed‐specific synthesis of hydroxylated fatty acids substrates was investigated. Functional expression of a Δ^12^‐oleate hydroxylase (CpFAH) from *Claviceps purpurea* (Meesapyodsuk and Qiu, 2008) under the control of a seed‐specific promoter was previously shown to result in high levels of ricinoleic acid (12‐hydroxyoctadec‐cis‐9‐enoic acid) accumulation in Arabidopsis. CpFAH was therefore chosen to provide a source of such hydroxylated fatty acids in Camelina. CpFAH is active on substrates esterified to membrane phospholipids, and therefore, hydroxylated fatty acids would only be made available for both wax ester and TAG synthesis via endogenous acyl exchange, primarily from PC into the acyl‐CoA pool (Figure 1a; pathway 2). In this study, the level of 12‐OH‐18:1Δ^9^ accumulated in TAG ranged from 0.4% to 3% of total fatty acids (Table 1). To investigate our approach to modulating substrate supply and tailoring wax ester synthesis, a total of fourteen constructs were prepared containing selected FAR and WS genes in combination with either an acyl‐ACP thioesterase or CpFAH under the control of seed‐specific promoters (Figure 1b) and identified by the abbreviations listed in Figure 1c.

**Table 1:** Camelina seed fatty acid composition. Fatty acid methyl esters were analysed by GC‐FID and expressed as mol %. The transgenes present in the different lines are detailed in Figure 1

	10:0	12:0	14:0	16:0	18:0	18:1n9	18:2n6	18:3n3	20:0	20:1n11	20:2n6	22:0	22:1n9	24:1n9	18:1OH	Others
Wt	0.0 ± 0.0	0.0 ± 0.0	0.0 ± 0.0	6.0 ± 0.1	3.0 ± 0.1	13.2 ± 0.5	17.9 ± 0.0	33.1 ± 0.5	2.0 ± 0.1	14.9 ± 0.3	2.2 ± 0.1	1.5 ± 0.1	3.3 ± 0.1	0.8 ± 0.0	0.0 ± 0.0	2.2 ± 0.3
MaMaWt	0.0 ± 0.0	0.0 ± 0.0	0.0 ± 0.0	6.2 ± 0.2	4.1 ± 0.1	14.2 ± 0.4	14.4 ± 0.1	33.1 ± 0.5	2.9 ± 0.0	14.8 ± 0.4	1.5 ± 0.0	1.5 ± 0.0	3.6 ± 0.1	0.8 ± 0.1	0.0 ± 0.0	2.8 ± 0.0
MaMa10	0.7 ± 0.2	0.0 ± 0.0	0.1 ± 0.1	8.1 ± 0.4	4.4 ± 0.4	14.3 ± 1.1	26.7 ± 0.9	24.6 ± 1.8	2.2 ± 0.0	10.5 ± 0.7	1.7 ± 0.1	0.7 ± 0.1	2.5 ± 0.2	0.8 ± 0.0	0.0 ± 0.0	2.8 ± 0.1
MaMa12	0.0 ± 0.0	2.3 ± 0.0	0.8 ± 0.0	7.8 ± 0.4	3.7 ± 0.1	12.7 ± 0.4	26.1 ± 0.8	24.4 ± 0.4	2.0 ± 0.1	11.8 ± 0.6	1.9 ± 0.1	0.8 ± 0.1	2.5 ± 0.2	0.8 ± 0.1	0.0 ± 0.0	2.4 ± 0.1
MaMa14	0.0 ± 0.0	0.0 ± 0.0	14.0 ± 1.1	15.1 ± 0.9	2.8 ± 0.0	8.5 ± 0.1	11.6 ± 0.3	27.1 ± 0.8	2.3 ± 0.1	10.2 ± 0.9	1.2 ± 0.1	1.1 ± 0.1	3.2 ± 0.4	0.6 ± 0.1	0.0 ± 0.0	2.3 ± 0.1
MaMaOH	0.1 ± 0.1	0.0 ± 0.0	0.0 ± 0.1	7.2 ± 1.1	3.9 ± 0.9	12.2 ± 1.3	22.4 ± 0.5	26.7 ± 2.4	2.7 ± 0.1	13.8 ± 1.0	2.1 ± 0.2	1.0 ± 0.1	3.6 ± 0.6	0.8 ± 0.2	0.4 ± 0.5	3.1 ± 0.2
MoMaWt	0.0 ± 0.0	0.0 ± 0.0	0.0 ± 0.0	5.6 ± 0.1	3.3 ± 0.1	14.4 ± 0.5	17.5 ± 0.2	31.2 ± 0.7	2.0 ± 0.1	16.6 ± 1.0	2.0 ± 0.0	1.3 ± 0.0	2.9 ± 0.2	0.8 ± 0.1	0.0 ± 0.0	2.3 ± 0.1
MoMa10	1.8 ± 0.3	0.3 ± 0.1	0.3 ± 0.1	6.4 ± 0.6	4.0 ± 0.5	12.6 ± 1.1	19.7 ± 1.3	26.8 ± 1.7	3.4 ± 0.1	12.7 ± 1.0	2.0 ± 0.3	1.1 ± 0.3	4.6 ± 0.4	1.0 ± 0.2	0.0 ± 0.0	3.3 ± 0.1
MoMa12	0.0 ± 0.1	1.0 ± 0.2	0.4 ± 0.0	6.5 ± 0.2	3.5 ± 0.1	11.0 ± 0.3	18.9 ± 0.0	28.9 ± 0.4	2.1 ± 0.0	17.7 ± 0.9	2.3 ± 0.1	1.2 ± 0.0	3.5 ± 0.2	1.0 ± 0.1	0.0 ± 0.0	2.2 ± 0.1
MoMa14	0.0 ± 0.0	0.0 ± 0.0	4.9 ± 0.6	10.8 ± 0.5	3.6 ± 0.1	10.3 ± 0.2	16.7 ± 0.2	27.5 ± 0.2	1.9 ± 0.0	15.8 ± 0.8	1.8 ± 0.1	1.1 ± 0.1	2.9 ± 0.2	0.8 ± 0.1	0.0 ± 0.0	2.0 ± 0.1
MoMaOH	0.0 ± 0.0	0.0 ± 0.0	0.0 ± 0.1	8.1 ± 0.3	7.8 ± 0.4	22.3 ± 0.4	18.2 ± 0.3	17.2 ± 0.5	2.7 ± 0.1	15.5 ± 0.6	0.8 ± 0.1	0.4 ± 0.0	1.4 ± 0.1	0.1 ± 0.1	3.0 ± 0.3	2.5 ± 0.1
AcMa14	0.0 ± 0.0	0.0 ± 0.0	2.1 ± 0.3	9.0 ± 0.3	3.7 ± 0.1	10.7 ± 0.2	18.2 ± 0.6	26.3 ± 0.5	2.1 ± 0.1	18.7 ± 0.7	2.1 ± 0.1	1.1 ± 0.1	3.2 ± 0.2	0.7 ± 0.1	0.0 ± 0.0	2.1 ± 0.1
TeMa14	0.0 ± 0.0	0.0 ± 0.0	10.1 ± 0.5	13.8 ± 0.3	3.6 ± 0.1	8.2 ± 0.2	20.7 ± 0.3	20.1 ± 0.6	2.5 ± 0.1	12.8 ± 0.1	1.8 ± 0.0	0.7 ± 0.0	3.0 ± 0.2	0.7 ± 0.0	0.0 ± 0.0	1.8 ± 0.1
MaTeWt	0.0 ± 0.0	0.0 ± 0.0	0.0 ± 0.0	6.6 ± 0.2	2.9 ± 0.1	9.4 ± 0.2	16.0 ± 0.4	36.6 ± 0.6	1.8 ± 0.0	15.3 ± 0.4	2.4 ± 0.1	2.0 ± 0.1	3.4 ± 0.2	0.9 ± 0.1	0.0 ± 0.0	2.8 ± 0.1
MaTe14	0.0 ± 0.0	0.0 ± 0.0	17.0 ± 0.1	17.8 ± 0.2	2.7 ± 0.0	5.8 ± 0.2	13.0 ± 0.3	25.7 ± 0.5	2.2 ± 0.1	8.1 ± 0.0	1.5 ± 0.0	1.1 ± 0.0	2.7 ± 0.2	0.6 ± 0.1	0.0 ± 0.0	1.7 ± 0.1

### Production of wax esters in seeds of Camelina

**Figure 2:**
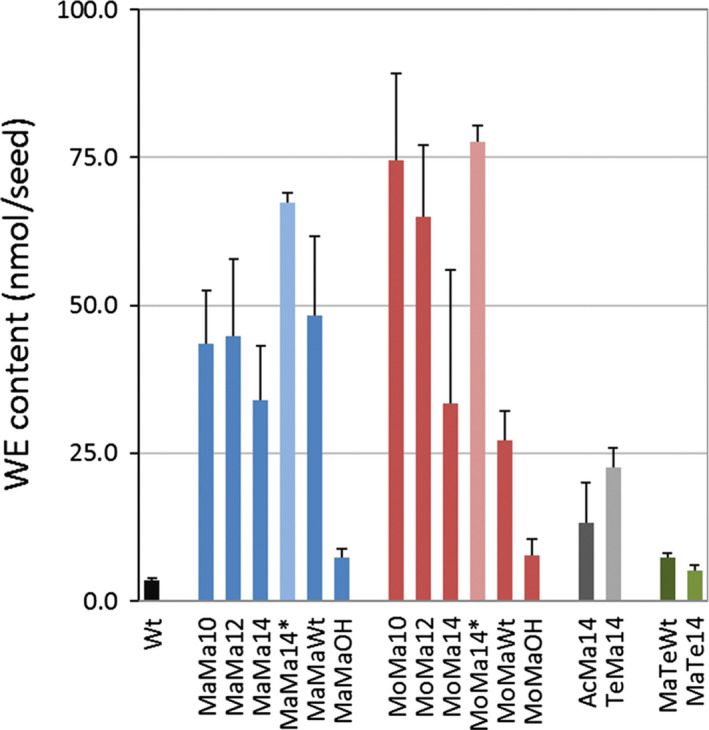
Wax ester content in the seeds of wild‐type and transgenic *Camelina sativa* lines expressing different combinations of wax biosynthetic and fatty acid modifying enzymes. This data shows the absolute WE content (in nmol/seed) in T2 seeds for all gene combinations and in T3 Seeds for MaMa14 and MoMa14 (*). Total lipid was extracted from 30 seeds and WE was separated from TAG and quantified by HPLC‐ELSD as described in the experimental procedures. For each construct the average WE content (±SE) was calculated from three independent transgenic lines.

Analysis of lipid extracts by HPLC‐ELSD from ten to fifteen independent T2 transgenic (thirty seeds per analysis) events for each gene combination indicated the successful production of wax esters (Figure 2). A very small amount of ester lipids (3.6 nmol seed^−1^) was detected in wild‐type (Wt) seeds, and this level was established as the baseline for successful wax ester biosynthesis. The transgenic Camelina lines accumulated between 4.6 and 87.6 nmol seed^−1^ of wax esters—this represented the maximum and minimum levels observed across all events (see also mean accumulation levels in Table 2); with the lowest levels of accumulation measured in lines MaTe (WS and FAR) combinations (~6 nmol seed^−1^). Wax ester synthesis in combination with the production of hydroxy fatty acids yielded modest but greater levels than Wt (7.3 and 7.8 nmol seed^−1^; MaMa and MoMa, respectively), demonstrating the feasibility of this approach. Maximal amounts of wax ester synthesis occurred in combinations of MaMa and MoMa with acyl‐ACP thioesterases, namely MaMa12 (12:0‐ACP thioesterase) 44.8 nmol seed^−1^ and MoMa10 (10:0‐ACP thioesterase) 74.6 nmol seed^−1^. Further analysis of MaMa14 (67.4 nmol seed^−1^) and MoMa14 (77.6 nmol seed^−1^) T3 lines revealed very significant increases in the accumulation of wax esters (98% and 130%, respectively) compared to T2 seeds (Figure 2). Previous work with constructs containing FAR and WS genes from *M. aquaeolei* and *M. musculus* (Heilmann *et al*., 2012 and Iven *et al*., 2016; respectively) has demonstrated the efficacy of these enzymes for *in planta* wax ester synthesis, with a reported range in Camelina of 7–47 mg/g seed for combinations of *M. musculus*, *M. aquaeolei* and *Simmondsia chinensis* FAR and WS. Here, total wax ester yields in seeds of Camelina ranged between 9.8 and 34.3 mg/g seed for MoMa(Wt) and MaMa(Wt) combinations, respectively (Fig. S1a). Because of significant variation in seed weight observed in this study (as discussed below), the quantity of WE produced per seed illustrates heterologous enzyme activity in each transgenic line more faithfully than WE quantity per g of seeds which is a function of seed weight and displayed significantly different patterns (e.g. for MoMa10, MoMa12 and T3 MoMa14 lines; Figures 2 and S1a). The addition of acyl‐ACP thioesterase activity generated maximal levels of 55.9 nmol seed^−1^ in MaMa12 and 87.6 nmol seed^−1^ in MoMa10. The impact of acyl‐ACP thioesterase expression on wax ester accumulation, modifying substrate availability, varies depending on the substrate specificity of the WS. A comparison of MaMaWt (*M. hydrocarbonoclasticus* WS, *M. aquaeolei* FAR and endogenous Camelina thioesterase activity) with lines expressing either acyl 10:0‐ACP thioesterase, 12 or 14, shows no beneficial impact on total wax ester production. However, the same comparison of endogenous versus introduced acyl‐ACP thioesterase combinations in lines expressing a mouse WS gene demonstrated a substantial increase in average levels of wax ester accumulation, for example MoMaWt (27 nmol seed^−1^) and MoMa10 (74.6 nmol seed^−1^). The mouse wax synthase has been suggested to prefer shorter‐chain (10:0, 12:0 and 14:0) acyl‐CoA species (Cheng and Russell, 2004), and this may contribute to the outcomes observed in this study. Unlike many FARs, the *M. aquaeolei* FAR has substantial activity with diverse substrates of different carbon chain length (C10 to C20), unsaturation and other substitutions (Hofvander *et al*., 2011) although this wide range was not fully reflected in our data. The *M. aquaeolei* FAR has reportedly the highest activity with 18:1‐CoA, and although capable of using ricinoleoyl‐CoA, fatty alcohol production is reduced by ~75%, which might explain the low wax ester yield in CpFAH‐expressing lines (MaMaOH and MoMaOH). The expression of a multifunctional *WS from A. calcoaceticus* ADP1 (a broad specificity bifunctional WS that also functions as an acyl‐CoA:diacylglycerol acyltransferase (DGAT)) and *Tetrahymena thermophila* WS in Camelina resulted in moderate levels of wax ester accumulation (Figure 2; AcMa14 and TeMa14). whereas expression of the bifunctional FAR gene (TtFARAT) from *Tetrahymena thermophila* generated only low levels of wax ester accumulation in combination with the *M. hydrocarbonoclasticus* WS (Figure 2; MaTeWt and MaTe14).

**Table 2:** The mean seed accumulation of WE for each different construct. Wax esters are expressed as nmol/seed, and SD indicated

Line	WE (nmol seed^−1^)
Wt	3.6 ± 0.3
MaMa10	43.5 ± 9.1
MaMa12	44.8 ± 13.0
MaMa14	34.0 ± 9.2
MaMaWt	48.3 ± 13.3
MaMaOH	7.3 ± 1.6
MoMa10	74.6 ± 14.7
MoMa12	65.1 ± 12.0
MoMa14	33.5 ± 22.5
MoMaWt	27.1 ± 5.1
MoMaOH	7.8 ± 2.8
AcMa14	13.2 ± 6.8
TeMa14	22.7 ± 3.2
MaTeWt	7.3 ± 0.7
MaTe14	5.2 ± 0.9
MaMa14_T3	67.4 ± 1.7
MoMa14_T3	77.6 ± 2.8

### Enzyme combinations influence wax ester composition

**Figure 3:**
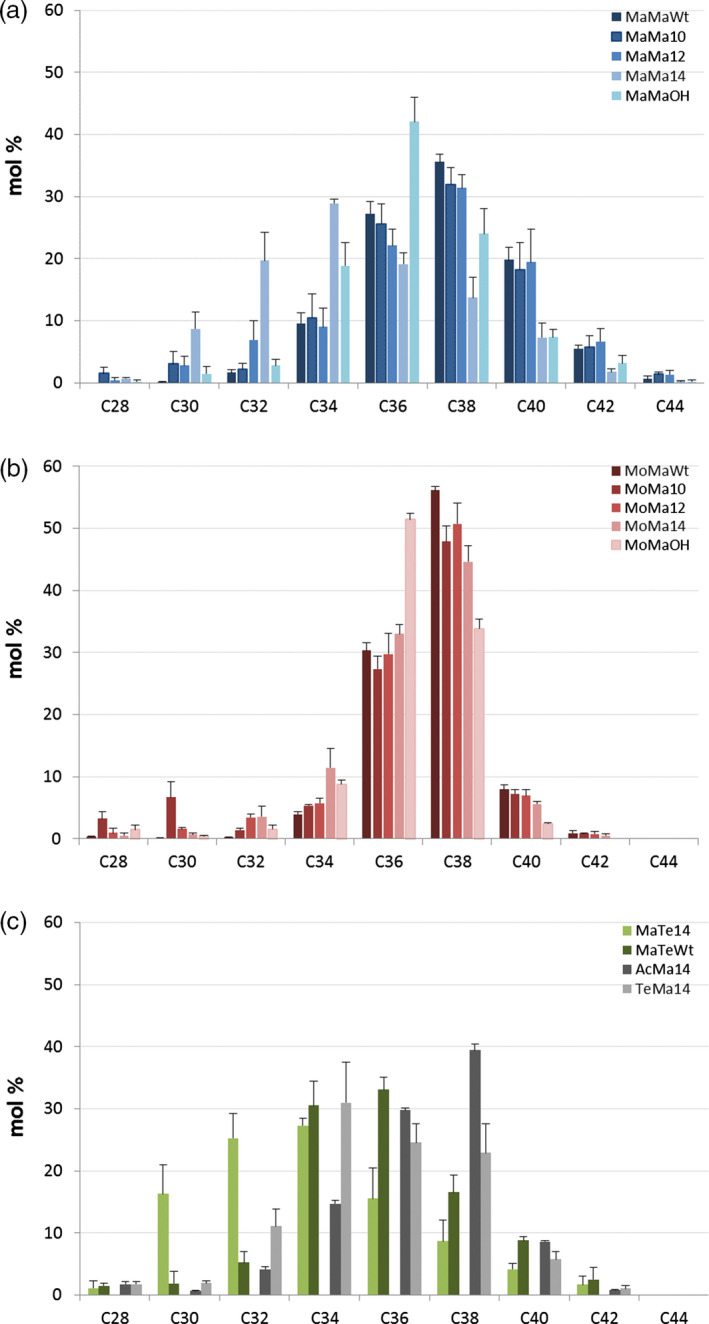
Wax ester composition in the seeds of T2 transgenic *Camelina sativa* lines expressing different combinations of wax biosynthetic and fatty acid modifying enzymes. Intact WEs were analysed by GLC as described in the experimental procedures and combined according to their total carbon number. The relative content (in mol %) in molecular species with chain lengths ranging from 28 to 44 carbons is shown for each gene combination. Transgenic lines expressing combinations of (a) *M. hydrocarbonoclasticus* WS and *M. aquaeolei* FAR or (b) *M. musculus* WS and *M. aquaeolei* FAR in different fatty acid backgrounds. (c) Lines expressing combinations of *M. hydrocarbonoclasticus* WS and *T. thermophila* FAR with *C. palustris* Thio14 or in Wt background, or combination of *A. calcoaceticus* or *T. thermophile* WSs with *M. aquaeolei* FAR and *C. palustris* Thio14. The average content (±SE) of three independent transgenic lines is shown for each gene combination.

**Figure 4:**
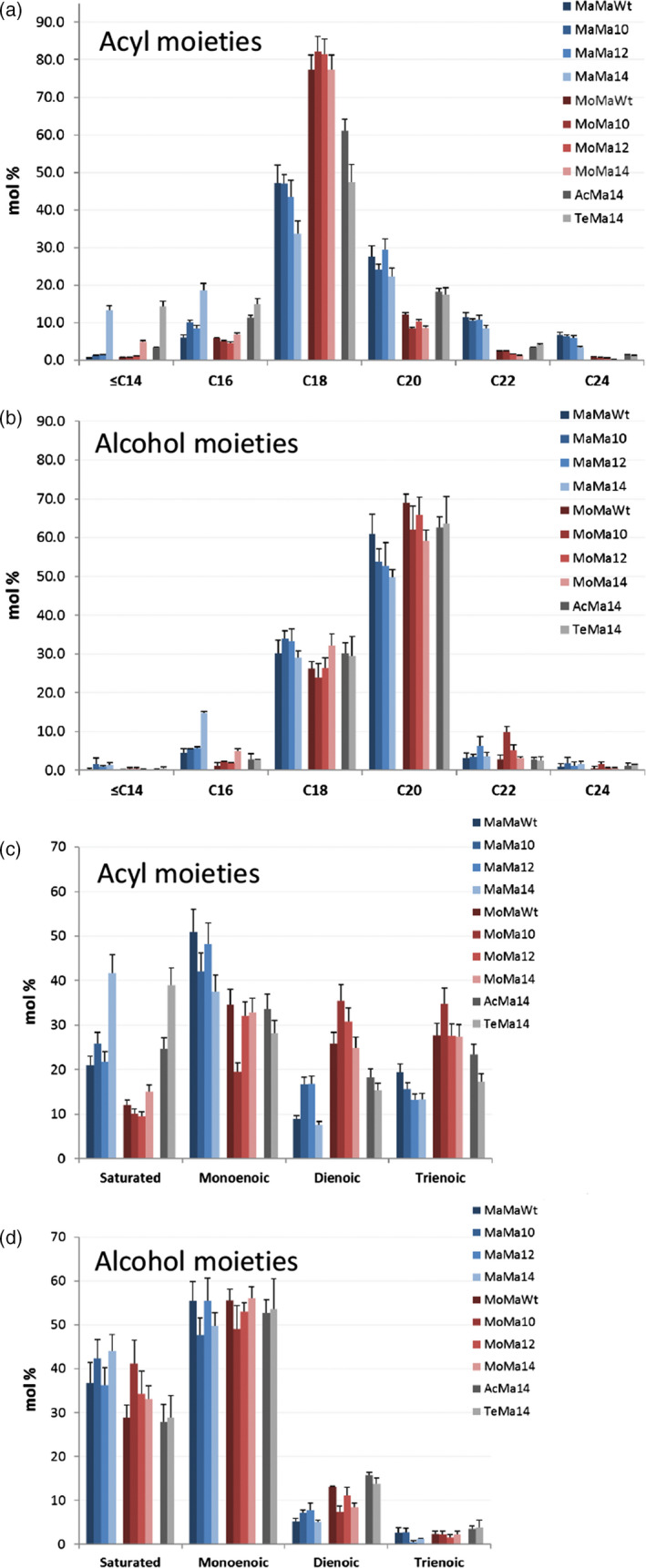
Composition (fatty acid and fatty alcohol) of wax esters produced in T2 seeds of transgenic *Camelina sativa* lines expressing different combinations of wax biosynthetic and fatty acid modifying enzymes. WE were hydrolysed and the resulting fatty acid and fatty alcohol moieties were derivatized, separated and quantified by GLC as described in the experimental procedure. This data shows the relative abundance of the wax ester moieties (in mol %) combining acyl chains according to the total carbon number (a, b) or the saturation degree (c, d) of the fatty acyl (a, c) or the fatty alcohol (b, d) moieties. Shown is the average content (±SE) of three independent transgenic lines for each gene combination.

Seed‐specific expression in Camelina of WS, FAR and thioesterase combinations was examined as a strategy to effectively tailor wax ester composition and thereby produce industrial oils with properties mimicking those of spermaceti oil. In this first iteration, it is necessary to understand how, in a boutique crop such as Camelina, different enzyme combinations effectively manipulate wax ester composition. Extraction and GC analysis of wax esters from the different transgenic lines showed specific compositional changes. The expression of *M. hydrocarbonoclasticus* WS and *M. aquaeolei* FAR (MaMa) in combination with endogenous or additional (C10:0, C12:0 and C14:0) acyl‐ACP thioesterases generated a broad distribution (mostly from C30 to C42) of wax esters (Figure 3a). Wax esters consisting of C36 and C38 (27% and 36%, respectively) molecular species predominated in MaMaWt lines. Additionally, the expression of heterologous acyl‐ACP thioesterases produced a shift in the distribution, reducing the accumulation of C36 and C38 species and, in particular with Thio14, increasing the contribution of C30–C34 and decreasing C36–C42. The expression of CpFAH generated a very specific (C34–C38; C36 in excess of 40%) wax ester composition. Then, purified WEs were transformed into fatty acid methyl esters and fatty alcohols by acid‐catalysed methanolysis, and analysis of the resulting acyl and alcohol moieties revealed how the use of specific thioesterases changed the composition. In the wax esters of MaMaWt C18/C20 (47% and 28%, respectively), saturated and monoenoic acyl and alcohol moieties predominate (Figure 4), with only small amounts (<10%) of C16/C22/C24. The expression of thioesterases 10, 12 and 14 resulted in a reduction in most fatty acyl moieties C18–C24, specifically with C18 declining from 47% to 34% in MaMa14 lines (Figure 4a). Conversely, in the same lines the contribution of shorter acyl moieties increased with C16 and species ≤C14 shifting from 6.1% and 0.6% to 18.6% and 13.3%, respectively. Remodelling of the alcohol moieties involved a reduction (61%–50%) in C20 species and an increase in C16 (5%–16%). The contribution of the predominant saturated or monoenoic alcohol moieties remained unchanged. Overall, the expression of *M. hydrocarbonoclasticus* WS and *M. aquaeolei* FAR generated wax ester species likely containing 18:1/18:1 (C36) and 18:1/20:1 (C38), and the expression of 14:0‐ACP thioesterase changed this to 14:0/18:1 (C32) and 14:0/20:1 (C34).

In an alternative iteration, the expression in Camelina of a mouse *M. musculus* WS and *M. aquaeolei* FAR (MoMa) generated a different and very specific wax ester composition of C36 (30%) and C38 (55%) species (Figure 3b). Despite earlier reported wide substrate specificity (Cheng and Russell, 2004), the mouse WS in Camelina preferentially incorporated C18/C20 alcohol moieties (27% and 65%, respectively; Figure 4) and monoenoic C18 acyl moieties (~80%; Figure 4) as reported by Heilmann *et al*. (2012) and Iven *et al*. (2016). The co‐expression of the (C10:0, C12:0 and C14:0) acyl‐ACP thioesterases in combination with mouse WS and *M. aquaeolei* FAR made little impact on the overall final wax composition. Analysis of the acyl‐CoA pool in the developing seeds of the transgenic MoMa Camelina lines indicated that thioesterase expression had resulted in substantial remodelling of the acyl‐CoA pool (Table S1); however, these substrates were not efficiently incorporated into WEs. Conversely, in the MaMa14 lines, the acyl‐CoA pool showed only a small increase in 14:0‐CoA, possibly reflecting the more efficient incorporation of that substrate into WEs by the *M. hydrocarbonoclasticus* WS. Only the expression of Thio14 (MoMa14) significantly reduced the accumulation of C38 (55%–45%) and concomitantly increased C34 content (5%–12%). Although accumulating much lower total levels of wax esters, the co‐expression of CpFAH with this construct combination (MoMaOH) produced a change in the ratio of C36 : C38 more pronounced than that observed in MaMaOH lines (Figure 3b; C36 52% and C38 33%). The incorporation of novel hydroxylated species in the wax ester pool is possible, as the two iterations have demonstrated (Figure 2); however, modification of the substrate pool requires careful selection of the WS and FAR activities for the successful high accumulations of these novel wax ester species. This is illustrated by the differential accumulation of Δ12‐OH‐C18:1 in TAGs and WEs in MaMaOH lines (0.4% and 4.7% of total FA) and MoMaOH lines (3% and <0.5% of total FAs, respectively; Table 1; data not shown). Overall, the wax ester composition resulting from the MoMa combination was very specific and most likely composed primarily of 18:1/20:1 (C38) and 18:1/18:1 (C36). However, it should be noted that in the absence of analysis of intact WEs, the precise acyl and alcohol composition of individual WEs remains to be proven. This is particularly the case for lines expressing the CpFAH hydroxylase.

**Figure 5:**
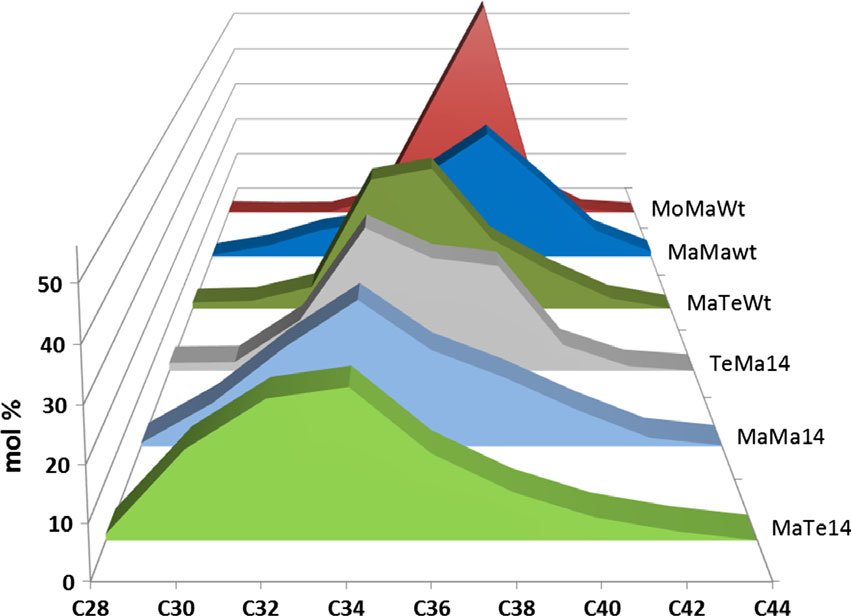
Comparison of wax ester profiles in T2 seeds of transgenic *Camelina sativa* lines expressing different wax biosynthetic and fatty acid modifying enzyme combinations. This figure illustrates the relative contribution (mol %) and distribution of WE molecular species combined according to their carbon chain length. The average of three independent transgenic lines is shown for each gene combination.

Changes in wax ester composition (and potential industrial utility) between the MaMa and MoMa WS/FAR enzyme combinations prompted examination of further combinations, for example MaTe14, AcMa14 and TeMa14 (Figure 1c). A comparison of the overall wax ester composition (Figure 5) clearly illustrates how different enzyme combinations can be used to generate very specific wax ester compositions. For example, in MaTe14 the combination of *M. hydrocarbonoclasticus WS, T. thermophile FAR* and *14:0‐ACP thioesterase* generated wax esters predominantly composed of C30–C36 (C34 27%; Figure 3c) whilst *A. calcoaceticus* WS, *M. aquaeolei* FAR and *14:0‐ACP thioesterase* (AcMa14) has a distribution of C34–C38 (C38 39%; Figure 3c) and *T. thermophile* WS, *M. aquaeolei* FAR and 14:0‐ACP thioesterase (TeMa14) of C32 to C38 (C34 30%; Figure 3c). For metabolic engineering, it is important to recognize how the different WS/FAR combinations generate wax esters with different compositions from the same substrate pool. Moreover, using different WS enzymes in combination with *M. aquaeolei* FAR illustrated how compositional changes can occur only in the acyl moiety of the wax esters; that is, TeMa14 incorporated greater amounts of shorter (<C16:0) saturated acyl moieties than AcMa14 (Figure 4a) with a similar alcohol profile (Figure 4b). Inversely, in MaTe14 lines the *T. thermophile* FAR more efficiently converted shorter‐chain acyl‐CoA than *M. aquaeolei* FAR resulting in a greater content in C12 and C14 fatty alcohols (8.8% C12 + C14; data not shown) and shorter WEs being produced (Figures 3c and 5). Finally, in MaMa and MoMa, combinations substrate supply and enzyme specificity combined to generate wax esters with changes in both the acyl and alcohol moieties.

### Properties of Camelina seed engineered for wax ester synthesis

**Figure 6:**
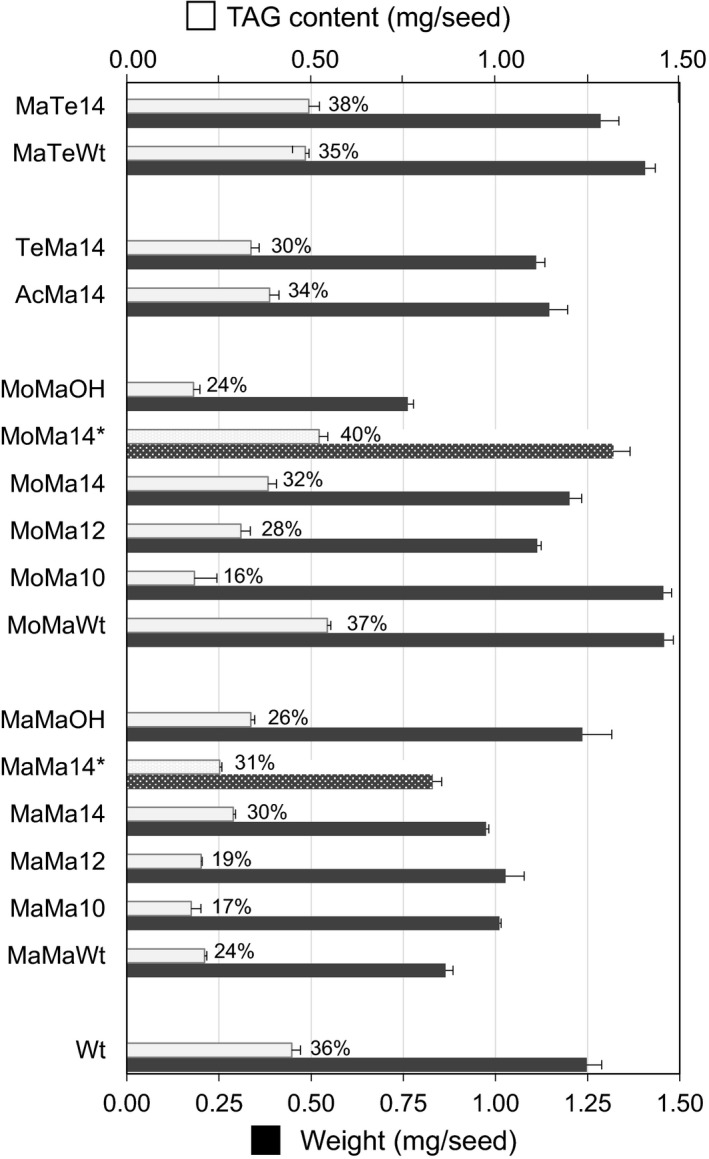
Seed weight and oil content in the seeds of wild‐type and transgenic *Camelina sativa* lines expressing different combinations of wax biosynthetic and fatty acid modifying enzymes. This data shows the absolute TAG content and the average seed weight (both in mg/seed) in T2 seeds for all gene combinations and in T3 Seeds for MaMa14 and MoMa14 (*). Average seed weight (±SD) was estimated by measuring the weight of 30 seed batches with 4 technical repeats for each transgenic line analysed. Relative seed oil content is shown as a percentage of seed weight (% w/w) at the top of the bars illustrating absolute TAG content.

Camelina seed TAGs typically contains approximately 20%–25% very long‐chain fatty acids (~15% of C20:1), low levels (<3.5%) of erucic acid (C22:1) and high levels of 18 carbon desaturated fatty acids such as α‐linolenic acid (30%–35% of 18 : 3). This was also reflected in the wax esters produced by the engineered Camelina lines, in which the acyl moieties predominantly contained C18 mono‐, di‐ and trienoic species, whereas the alcohol moieties typically contained C20 monoenoic species (Figure 4). A further aim of this study was to examine the influence seed wax ester synthesis has on TAG assembly, as recent research has indicated an associated reduction in oil content in transgenic lines (Zhu *et al*., 2016). Analysis of TAG accumulation in the seed of Camelina engineered for wax synthesis was determined by quantification of the fatty acid methyl esters using GC‐FID following separation of the TAG fraction by HPLC‐ELSD. The seed TAG content varied from 16% to 38% (36% in Wt; Figure 6) in transgenic lines. T2 lines accumulating the highest amounts of wax esters, for example MoMa10 and MaMa10 (Figure 2), had a correspondingly low TAG content (16% and 17%, respectively; Figure 6). However, certain iterations, for example MoMaWt (37% TAG) and MaMa14 (30%), retained wild‐type levels of seed oil accumulation. Furthermore, it should be noted that because of seed weight variation the relative TAG content in % (w/w) is not always representative of total seed oil content. For example, MoMaWt seeds contain 22% more oil than WT (0.544 and 0.447 mg/seed, respectively) in addition to ~15 μg (27 nmol) WE, hence bigger seeds. Those lines expressing CpFAH for the production of hydroxylated fatty acids were largely compromised in oil synthesis (24%–26% TAG), reflecting the difficulty of incorporating these novel species into wax ester and oil biosynthetic pathways and also the indirect impact of hydroxylated fatty acids on glycerolipid metabolism (van Erp *et al*., 2011). The composition of TAG in seed oil was also modified, typically as a result of acyl‐ACP thioesterase expression. Medium‐chain fatty acids could be identified in the seed oil (Table 1); for example, MaMa10, MaMa12 and MaMa14 contained 0.8%, 3.1% and 14% medium‐chain fatty acids (C10:0 to C14:0), whereas MoMa10, MoMa12 and MoMa14 accumulated 2.4%, 1.4% and 4.9%, respectively. The early termination of FAS by acyl‐ACP thioesterase expression also had an impact on the C18 content of TAG, typically 67% in Wt; this was reduced to 50% in the most effective MaMa14 iterations. As a general trend, seed oil accumulation declined with wax ester production and underwent compositional changes in response to the expression of different WS, FAR and acyl‐ACP thioesterase combinations. However, as discussed above this was not systematic and we have also identified lines in which both the TAG and the WE contents and were significantly increased compared to Wt Camelina seeds. Particularly, T3 MoMa14 lines displayed a 130% increase in seed WE content compared to T2 seeds (77.6 and 33.5 nmol/seed, respectively; Figure 2) but also contained 16.7% more TAG than WT seeds (0.522 and 0.447 mg/seed, respectively; Figure 6). This suggest that with the right enzyme combinations and tailored substrate pool compositions it is possible to engineer a wax biosynthetic path in the seed of Camelina that does not compete directly with endogenous lipid biosynthetic pathways. On average, there was a decline in seed weight with the expression of different gene combinations (Figure 6); however, a consistent pattern did not emerge. For example, of the high accumulating wax ester lines MaMa10 had a reduction in seed weight, whilst MoMa10 increased in seed weight. However, any difference in seed weight or oil content did not translate into any germination phenotype or detectable plant developmental defect. All Camelina T2 and T3 seeds germinated at similar rates and grew into plants indistinguishable from wild type.

### Towards the development of a new method for pilot‐scale extraction and separation of wax esters from seed oil

Seed oil WEs and TAGs can easily be extracted and separated in the laboratory using simple solvent extraction and separation by chromatography either on TLC or by HPLC. However, one aspect which will determine the economic viability of WEs produced in seeds as an alternative to nonrenewable sources is the ability to simply and cheaply extract them and separate them from TAGs at an industrial scale. There has been only one report suggesting that very long‐chain wax esters (C42–C48) produced in Camelina seeds could be at least partially separated from the TAGs by chilling the oil to 4 °C. This procedure called winterization leads to the formation of a solid fraction enriched in WEs that can be partially separated from the liquid oil (Zhu *et al*., 2016). However, this technique relies on WE species with melting points higher than that of seed oil TAGs and is therefore likely to be more challenging with shorter‐chain WE as the ones present in spermaceti (typically C28–C40) and the lines generated in this study. To test the possibility to efficiently extract and purify a sufficient amount of spermaceti‐like WEs from Camelina seeds for performance testing, a pilot‐scale experiment was conducted using the MaMa14 line described above. Two T3 MaMa14 lines were selected for their relatively high WE content (47 and 30 nmol/seed or 30 and 20 mg WE/g seed, respectively). Approximately 1200 plants were cultivated in containment glass houses, yielding 6.9 kg of T4 seeds. After pressing and hexane extraction 2.04 kg of oil was recovered (29.6%, w/w) and the WE concentration was estimated by HPLC at 25 mg/g oil corresponding to over 50 g of WE that could be potentially extracted (Table S2). This amount of WE was considered sufficient for performance testing as a lubricant therefore purification was undertaken.

**Figure 7:**
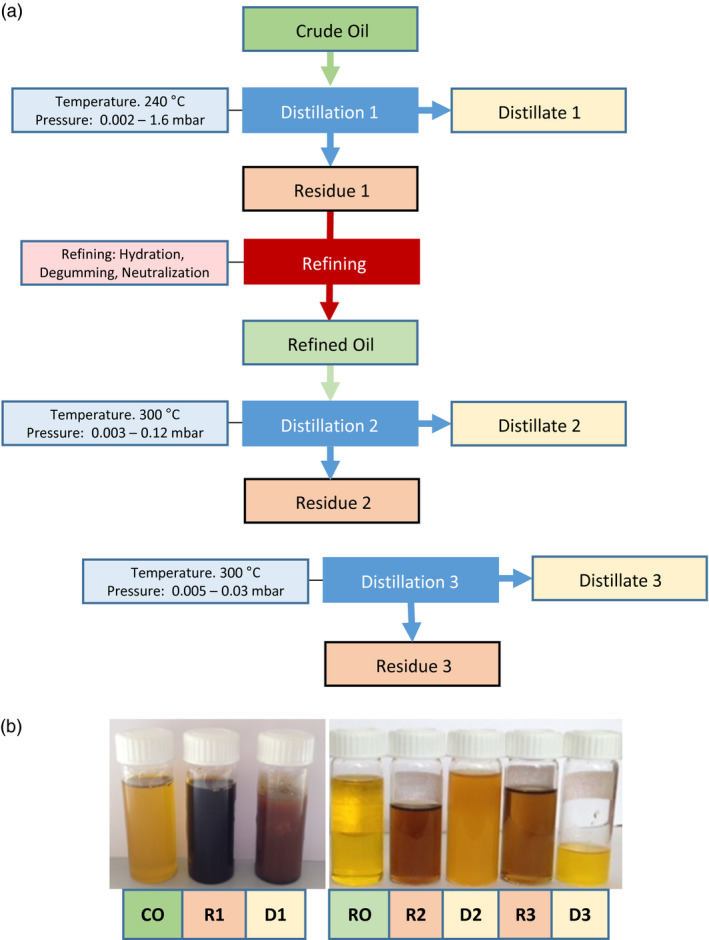
(a) Schematic overview of the Molecular Distillation (MD) and refining steps tested for purification of wax ester form Camelina seed oil. (b) Camelina seed oil fractions before and after refining and MD steps. CO, crude oil after cold pressing and solvent extraction; RO, refined oil after hydration, degumming and neutralization; D1‐3, distillates recovered; R1‐3, oil residues after MD steps.

WEs were initially purified using molecular distillation (MD) (Figure 7a), a procedure that can be used at both laboratory and industrial scale for purification of heat sensitive compounds. MD has been used to purify omega‐3 fatty acids (EPA and DHA) from fish oil (Bergeron *et al*. 2012) but also tocotrienols and tocopherols from palm and rapeseed oil deodorizer distillates, respectively (Jiang *et al*., 2006; Posada *et al*., 2007). MD presents many advantages such as the absence of a requirement for an organic solvent and reduced or no degradation of compounds due to high vacuum. Under high‐vacuum conditions, the boiling point of the components to be separated is 200–300 °C lower than at atmospheric pressure (see Table S4a, b), allowing separation of high molecular weight compounds with minimal degradation. Therefore, it was decided to test the efficiency of this method for separating WEs from TAGs using different experimental conditions (Figure 7a). As described in the Supp. Methods and Figs S1–S6, this method can be used for efficient extraction of nondegraded WEs from refined oil (over 80% recovery; Table S2). However, due to codistillation with short‐chain saturated TAG molecular species, only a moderate two fold enrichment was possible.

Winterization is a process commonly used industrially to remove components with high melting point (e.g. waxes) from vegetable oils such as sunflower, rice bran and cotton seed oil. Winterizing mainly consists of cooling the oil gradually and filtering it at a low temperature using special filters. As previous work has shown that long‐chain wax ester could be solidified and partially separated from Camelina seed oil (Zhu *et al*., 2016), the methods was tested for the purification of spermaceti‐like medium‐chain WEs. This method was less successful than molecular distillation as we were only able to recover less than 20% of WE present in the refined oil fraction (Table S3). However, the WE recovered in the crystallized fraction was heavily enriched in saturated species (80% of total WE content) suggesting that recovery could be greatly improved by saturating the oil by hydrogenation, a procedure easily and cheaply used industrially.

## Conclusion

In this study, the seed‐specific expression in Camelina of WS, FAR and thioesterase combinations was examined as a strategy to effectively tailor wax ester composition and thereby produce industrial oils with properties mimicking those of spermaceti oil. A comparison of the overall WE compositions generated illustrated how different enzyme combinations can yield very specific wax ester compositions. Depending on the enzymes used, compositional changes occurred in either or both the fatty acyl and the fatty alcohol moiety of the wax esters, although it is still (frustratingly) not possible to accurately predict the performance of a heterologous enzyme *in planta* (Napier *et al*., 2014). As a general trend, seed weight and oil content declined with wax ester production and TAG underwent compositional changes in response to the expression of different WS, FAR and fatty acid modifying enzyme combinations. However, a consistent pattern did not emerge and differences in seed weight or oil content did not translate into any germination phenotype or obvious plant developmental defect. Interestingly, we also identified lines producing bigger seeds in which both the TAG and the WE contents were significantly increased suggesting that, given the right enzyme combinations and tailored substrate pool compositions, it is possible to engineer wax biosynthetic pathways in Camelina seeds that do not compete directly with TAG synthesis. Finally, preliminary purification experiments suggest that it should be possible to develop a method for the efficient extraction and enrichment of WE from Camelina seed oil, and this is currently being investigated. This method will likely involve a sophisticated combination of biorefining techniques including both MD and winterizations steps.

## Experimental procedures

### Plant material and growth conditions

*Camelina sativa* (Camelina) were grown in a controlled‐environment chamber at 23 °C day/18 °C night, 50%–60% humidity, and kept on a 16‐h, 250 μmol m^−2^ s^−1^, photoperiod (long day).

### Generation of transgenic plants

Transgenic Camelina lines were generated as previously described with minor modifications (Lu and Kang, 2008; Ruiz‐Lopez *et al*., 2014). The designed vectors were transferred into *Agrobacterium tumefaciens* strain AGL1. Camelina inflorescences were immersed into the Agrobacterium suspension for 30 s without applying any vacuum. Transgenic seeds were identified by visual screening for DsRed activity. Seeds harvested from transformed plants were illuminated by a green LED flashlight. Fluorescent seeds were visualized using red‐lens filter.

### Vector construction

Fourteen constructs (Figure 1 C) were built using the Gateway recombination system (Invitrogen, Paisley, UK) as previously described in Ruiz‐Lopez *et al*. (2015). Respective genes were inserted as NcoI/PacI or Asc/PacI fragments into the promoter/terminator cassettes and then moved into pENTRY vectors. All open reading frames for fatty acid acyl‐CoA reductases, wax synthases and thioesterases or hydroxylases were resynthesized (GenScript Corporation, Piscataway, NJ) and codon‐optimized for expression for Camelina. The destination vector contained a DsRed gene as the selection marker, driven by the constitutive CsVMV promoter. All constructs contained two or three expression cassettes where each gene was under control of the conlinin1 promoter (pCnl) (Truksa *et al*., 2003) and linked to a terminator region of OCS, octopin synthase gene of *Agrobacterium tumefaciens*. In particular, MaMaWt construct contained a *M. aquaeolei fatty acyl reductase gene* (Hofvander *et al*., 2011) *and a M. hydrocarbonoclasticus wax synthase gene (GenBank accession* *ABO21021* *)*. MaMa10, MaMa12, MaMa14 and MaMaOH constructs shared the same FAR and WS genes than MaMaWt, but they also included a *Cuphea hookeriana 8:0‐ and 10:0‐ACP specific thioesterase gene* (Dehesh *et al*., 1996a) *or* a *Umbellularia californica* 12:0‐ACP specific thioesterase gene (Voelker *et al*., 1992) or a 14:0‐ACP specific thioesterase gene from *C. palustris* (Dehesh *et al*., 1996b), or a *Claviceps purpurea* fatty acid hydroxylase gene (Meesapyodsuk and Qiu, 2008), respectively. Additionally, MoMaWt, MoMa10, MoMa12, MoMa14 and MoMaOH constructs included the same genes as the constructs detailed above, but they contained a wax synthase gene from *M. musculus* (Cheng and Russell, 2004) replacing the *M. hydrocarbonoclasticus* WS. In addition, AcMa14 and TeMa14 constructs also comprised of three expression cassettes including (i) a FAR gene from *M. aquaeolei*, (ii) a 14:0‐ACP specific thioesterase gene from *C. palustris* and (iii) AcMa14 included a WS gene from *A. calcoaceticus* (Kalscheuer and Steinbuchel, 2003), but TeMa14 included a WS gene from *T. thermophile* (Biester *et al*., 2012). Finally, MaTe14 and MaTeWt constructs were built with (i) a WS gene from *M. hydrocarbonoclasticus* and (ii) a FAR gene from *T. thermophile* (Dittrich‐Domergue *et al*., 2014), both flanked by conlinin1 promoters and OCS terminators as in the above constructs. Additionally, MaTe14 construct also included a 14:0‐ACP specific thioesterase gene from *C. palustris*.

### Acyl‐CoA profiling

Camelina seeds (ten seeds, 28 DAF) were harvested and frozen in liquid nitrogen and extracted after Larson and Graham (2001) for reverse‐phase LC with electrospray ionization tandem mass spectrometry (multireaction monitoring) in positive ion mode. LC‐MS/MS MRM analysis followed the methods described by Haynes *et al*. (2008). (Agilent 1200 LC system; Gemini C18 column, 2 mm inner diameter, 150 mm with 5‐mm particles). For the purpose of identification and calibration, standard acyl‐CoA esters with acyl chain lengths from C14 to C20 were purchased from Sigma as free acids or lithium salts.

### Lipid extraction, separation and quantification by HPLC‐ELSD

Wax esters and TAG were extracted following the Hara and Radin (1978) method with some modifications. Thirty Camelina seeds (approximately 20 mg) were heated in 0.8 mL of isopropanol at 85 °C for 10 min. Then, 1.2 mL of hexane was added and seeds were homogenized using a mortar and pestle. Anhydrous sodium sulphate was added to remove any residual moisture and to facilitate the separation of lipid phase. The homogenate was centrifuged, supernatant collected and the pellet re‐extracted with hexane : isopropanol (7 : 2, by vol). Both extracts were pooled, evaporated and diluted in 200 μL of hexane. An evaporative light scattering detection (ELSD, Agilent 1200 Infinity Evaporative Light Scattering Detector)‐based high‐performance liquid chromatography (HPLC, Agilent 1100 LC system) method was used based on the Aragón *et al*. (2011) method with some modifications. The separation was carried out on a 250 mm × 4 mm i.d. column packed with modified silica (Lichrospher 5 μm Si 60; Phenomenex) maintained at 25 °C. The initial composition of the eluent (95 : 5 hexane : ethyl acetate; v/v) at a flow rate of 0.5 mL/min was maintained for 8.5 min. Then, the flow was increased to 0.7 mL/min and the gradient was varied to reach 50% ethyl acetate within 0.5 min and maintained for 5 min. To assure the complete elimination of impurities, the gradient was set to 80% ethyl acetate and it was maintained for 2 min. For the analysis of the samples, 100 μL of the oil sample prepared as described above was injected. The ELSD was maintained at 40 °C throughout. The nebulizer (nitrogen) gas pressure was set at 3.5 bar and the detector gain was set at 2. WE and TAG peaks were identified by comparison with standards (Sigma‐Aldrich, UK), and they were collected at their specific elution times using a fraction collector. WE quantification was performed by area comparison of C28:0, C30:0, C32:0, C34:0, C36:0, C38:0 and C40:0 standards from Sigma‐Aldrich. To adjust for the effects of the nonlinear response curve of the ELSD detector, calibration curves for WEs were generated using C28 to C44 commercially available standards. The calibration was made with lipid standard mixtures as close as possible in composition to those observed in the experimental samples.

### Wax ester analysis

Intact wax esters were separated and quantified by GLC with an Agilent 6890 gas chromatograph (Palo Alto, CA), using a HP‐1 ms capillary column from Agilent J&W (30 m, 0.25 mm, 0.25 μm). Helium was used as carrier gas, and injector and detector temperatures were 280 and 325 °C, respectively. Splitless injection was used, with a flow rate set at 1.5 mL/min. After injection at 70 °C, the oven temperature was raised to 200 °C at a rate 50 °C min^−1^, then to 325 °C at a rate of 3 °C min^−1^, and held constant for 10 min. The detector used was a flame ionization detector. Wax esters peaks were identified by comparing their retention times with those of authentic standards (C28–C46; Nu‐Chek Prep Inc, Elysian, MN, USA.) and quantified using calibration curves essentially as described in Razeq *et al*. (2014).

### Analysis of fatty acid composition in WE and TAG

Total fatty acids from wax esters and TAG were transmethylated by heating the samples at 80 °C for 2 h with 2 mL of a solution containing methanol/toluene/dimethoxypropane/H_2_SO_4_ (66 : 28 : 2 : 1 by volume) as previously described (Ruiz‐Lopez *et al*., 2015). Methyl ester derivatives of total fatty acids were extracted using hexane, and they were analysed by GC‐FID and confirmed by GC‐MS. Quantification of fatty acids from TAG was performed using tri‐heptadecanoyl glycerol (Sigma‐Aldrich) as an internal standard. Data are presented as representative numbers derived from three replicated analyses.

### Fatty alcohol separation and analysis

Fatty alcohols were separated of FAMES using SPE silica gel 60 columns (Merck). The mixture of FAMEs and fatty alcohols was dissolved in hexane and poured on the column prepared in hexane. FAMEs were eluted after adding 6 mL of hexane : diethyl ether (95 : 5, v/v). Fatty alcohols were eluted by adding 6 mL of hexane:ethyl acetate (6/1, v/v). Fatty alcohols were silylated by adding excess of BSTFA and 1% TMCS and heating the samples at 80 °C for 1 h prior to analysing them by GC, as below.

## Conflict of interest

All authors declare that they have no conflict of interest.

## Supporting information


**Figure S1** (a) Wax ester content in the seeds of wild‐type and transgenic Camelina sativa lines expressing different combinations of wax biosynthetic and fatty acid modifying enzymes. (b) Wax ester profile in the seeds of T3 transgenic lines expressing either MaMa14 (left panel) or MoMa14 (right panel) enzyme combinations.
**Figure S2** Wax ester composition of distillate and residue fractions after molecular distillation of T3 MaMa14 Camelina seed oil.
**Figure S3** GC‐FID analysis of wax esters in distillate and residue fractions after molecular distillation of T3 MaMa14 Camelina seed oil.
**Figure S4** Comparison of the wax ester composition in T3 MaMa14 Camelina seed oil and in distillate 2 fraction.
**Figure S5** Fatty acid composition of TAG in distillate and residue fractions after molecular distillation of T3 MaMa14 Camelina seed oil.
**Figure S6** Oil winterization.

**Table S1** Acyl‐CoA profiling of developing Camelina Seeds
**Table S2** Molecular distillation results
**Table S3** Oil winterisation results
**Table S4a** Physico‐chemical properties of saturated wax esters
**Table S4b** Physico‐chemical properties of unsaturated wax esters

Supplementary Legends
