# Supplementary material for: Tailoring the composition of novel wax esters in the seeds of transgenic Camelina sativa through systematic metabolic engineering
Source: Plant Biotechnol J. 2017 Feb 2;15(7):837–49. doi: 10.1111/pbi.12679 (PMC5466440; doi:10.1111/pbi.12679)
Supplement: Supplementary file 1 — Figure S1 (a) Wax ester content in the seeds of wild‐type and transgenic Camelina sativa lines expressing different combinations of wax biosynthetic and fatty acid modifying enzymes. (b) Wax ester profile in the seeds of T3 transgenic lines expressing either MaMa14 (left panel) or MoMa14 (right panel) enzyme combinations. Figure S2 Wax ester composition of distillate and residue fractions after molecular distillation of T3 MaMa14 Camelina seed oil. Figure S3 GC‐FID analysis of wax esters in distillate and residue fractions after molecular distillation of T3 MaMa14 Camelina seed oil. Figure S4 Comparison of the wax ester composition in T3 MaMa14 Camelina seed oil and in distillate 2 fraction. Figure S5 Fatty acid composition of TAG in distillate and residue fractions after molecular distillation of T3 MaMa14 Camelina seed oil. Figure S6 Oil winterization. [file PBI-15-837-s002.pptx]

## Slide 1
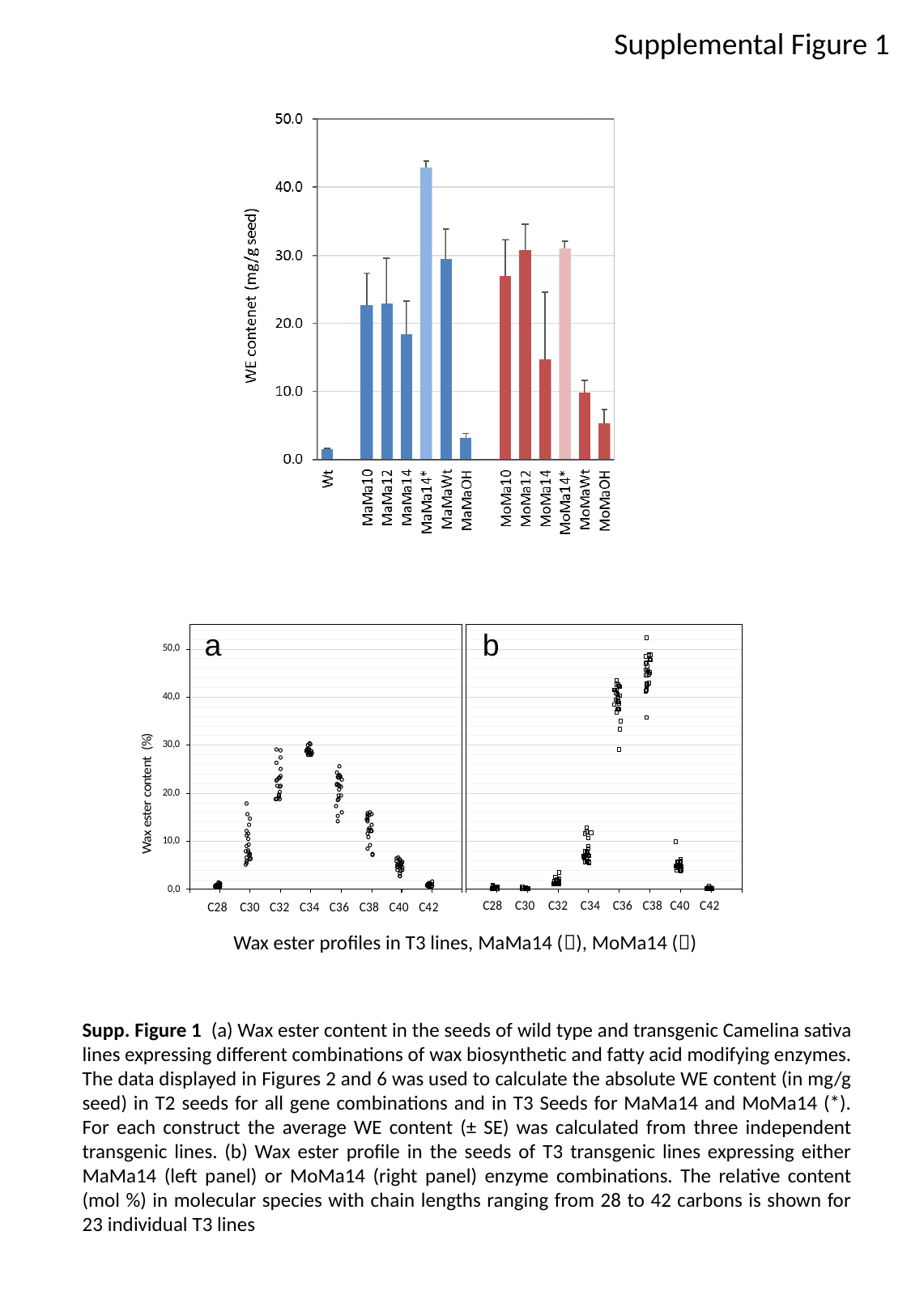

Supplemental Figure 1
a
b
Wax ester profiles in T3 lines, MaMa14 (), MoMa14 ()
Supp. Figure 1 (a) Wax ester content in the seeds of wild type and transgenic Camelina sativa lines expressing different combinations of wax biosynthetic and fatty acid modifying enzymes. The data displayed in Figures 2 and 6 was used to calculate the absolute WE content (in mg/g seed) in T2 seeds for all gene combinations and in T3 Seeds for MaMa14 and MoMa14 (*). For each construct the average WE content (± SE) was calculated from three independent transgenic lines. (b) Wax ester profile in the seeds of T3 transgenic lines expressing either MaMa14 (left panel) or MoMa14 (right panel) enzyme combinations. The relative content (mol %) in molecular species with chain lengths ranging from 28 to 42 carbons is shown for 23 individual T3 lines

## Slide 2
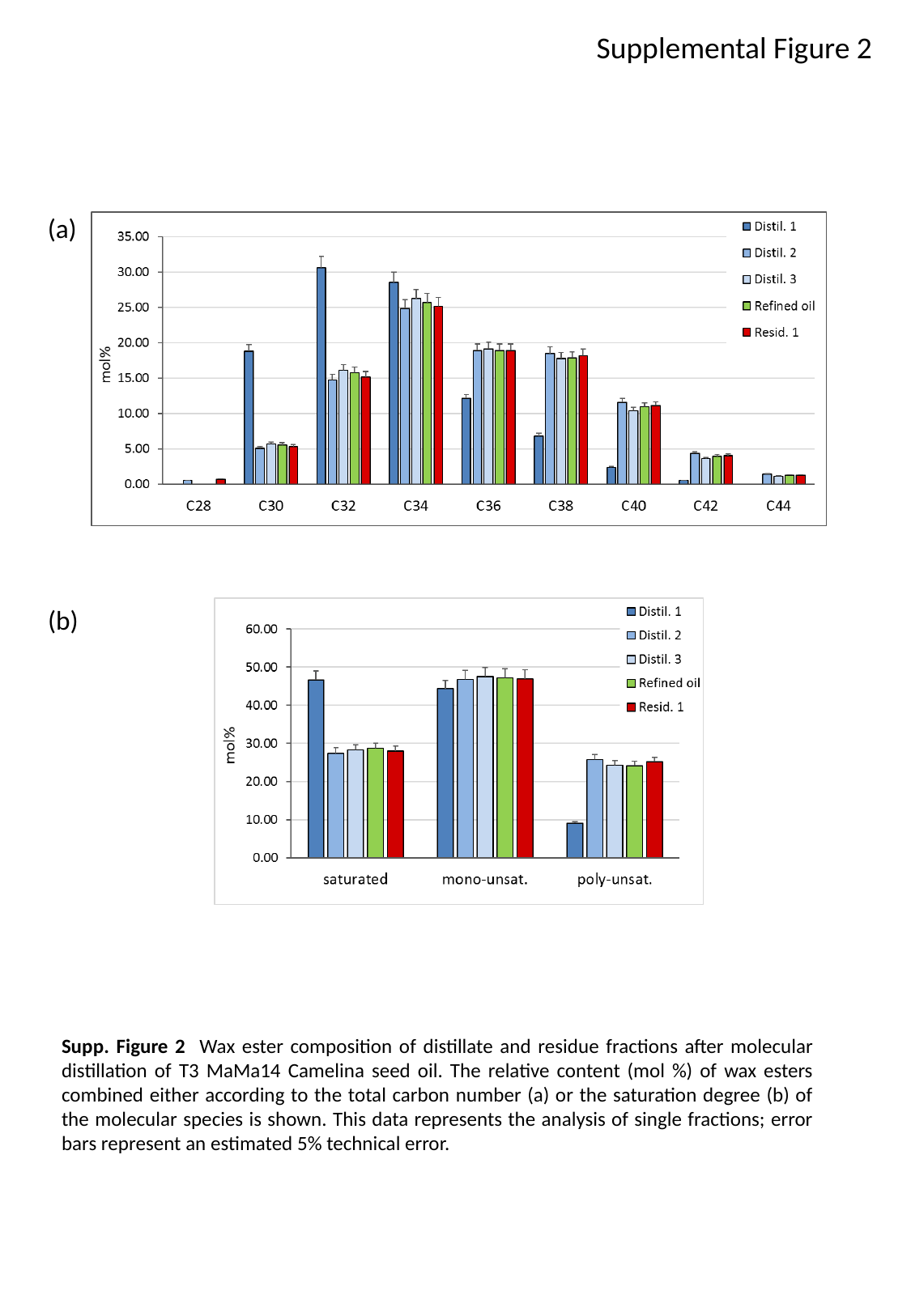

Supplemental Figure 2
(a)
(b)
Supp. Figure 2 Wax ester composition of distillate and residue fractions after molecular distillation of T3 MaMa14 Camelina seed oil. The relative content (mol %) of wax esters combined either according to the total carbon number (a) or the saturation degree (b) of the molecular species is shown. This data represents the analysis of single fractions; error bars represent an estimated 5% technical error.

## Slide 3
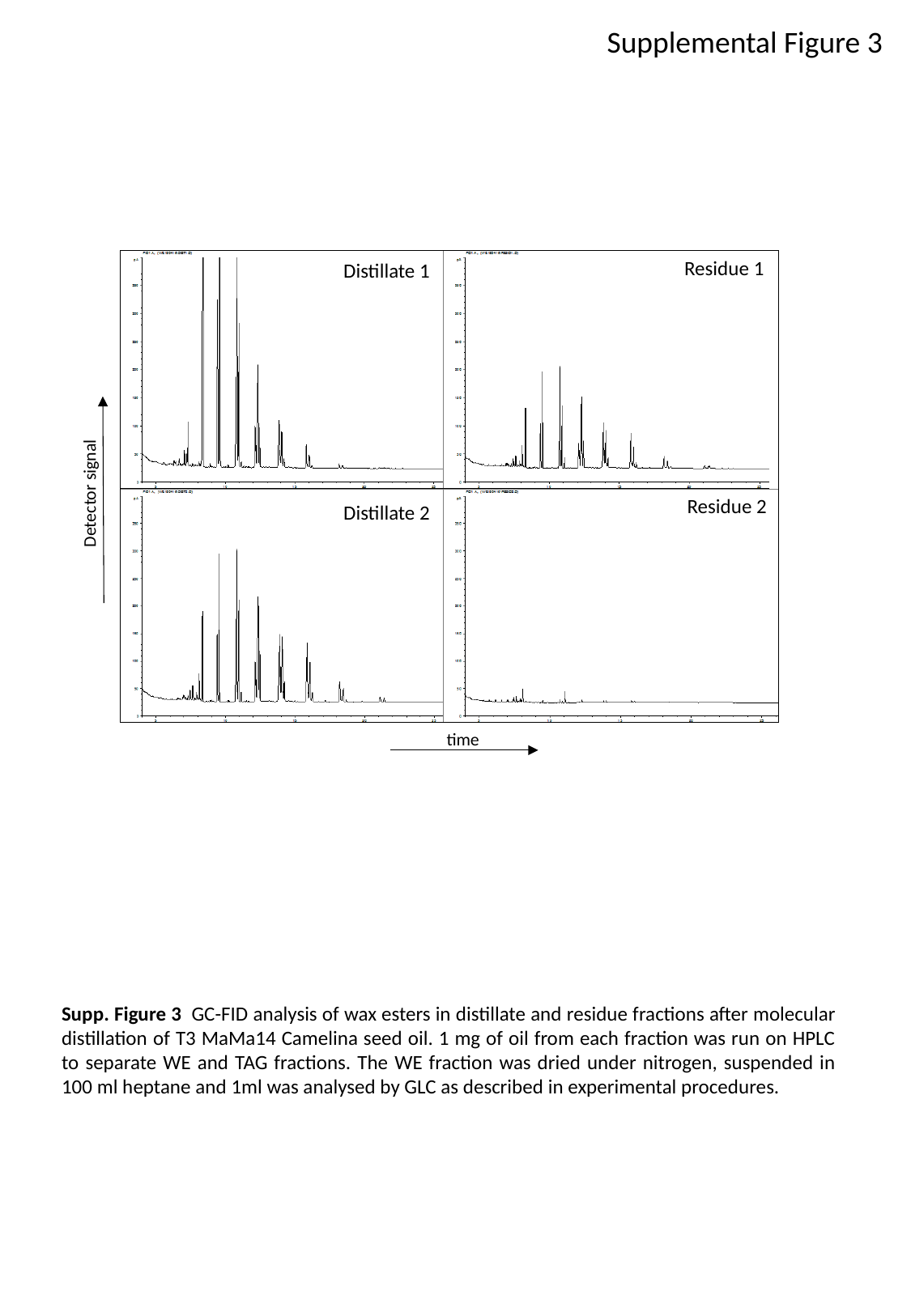

Supplemental Figure 3
Residue 1
Distillate 1
Detector signal
Residue 2
Distillate 2
time
Supp. Figure 3 GC-FID analysis of wax esters in distillate and residue fractions after molecular distillation of T3 MaMa14 Camelina seed oil. 1 mg of oil from each fraction was run on HPLC to separate WE and TAG fractions. The WE fraction was dried under nitrogen, suspended in 100 ml heptane and 1ml was analysed by GLC as described in experimental procedures.

## Slide 4
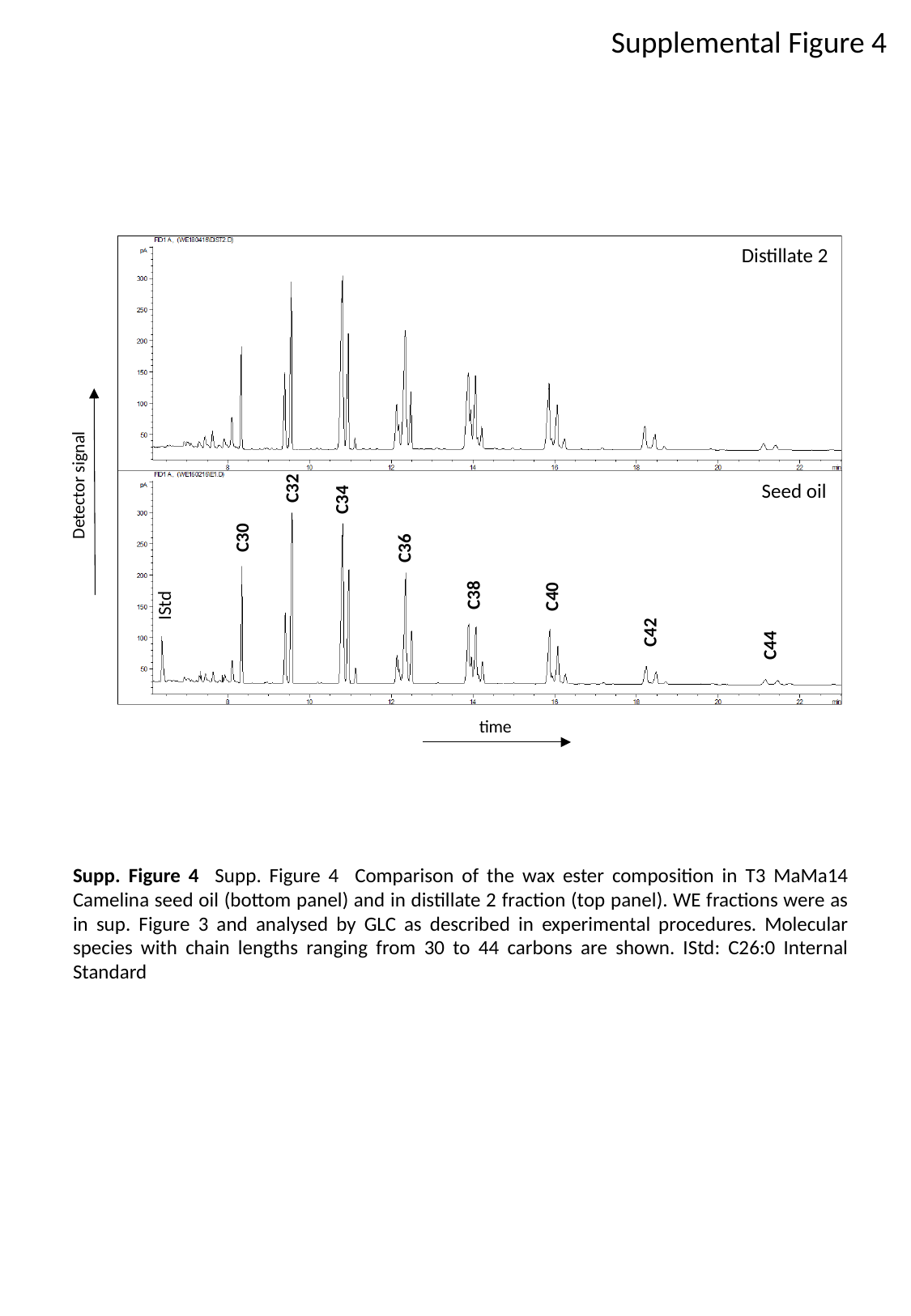

Supplemental Figure 4
Distillate 2
Detector signal
C32
Seed oil
C34
C30
C36
C38
C40
IStd
C42
C44
time
Supp. Figure 4 Supp. Figure 4 Comparison of the wax ester composition in T3 MaMa14 Camelina seed oil (bottom panel) and in distillate 2 fraction (top panel). WE fractions were as in sup. Figure 3 and analysed by GLC as described in experimental procedures. Molecular species with chain lengths ranging from 30 to 44 carbons are shown. IStd: C26:0 Internal Standard

## Slide 5
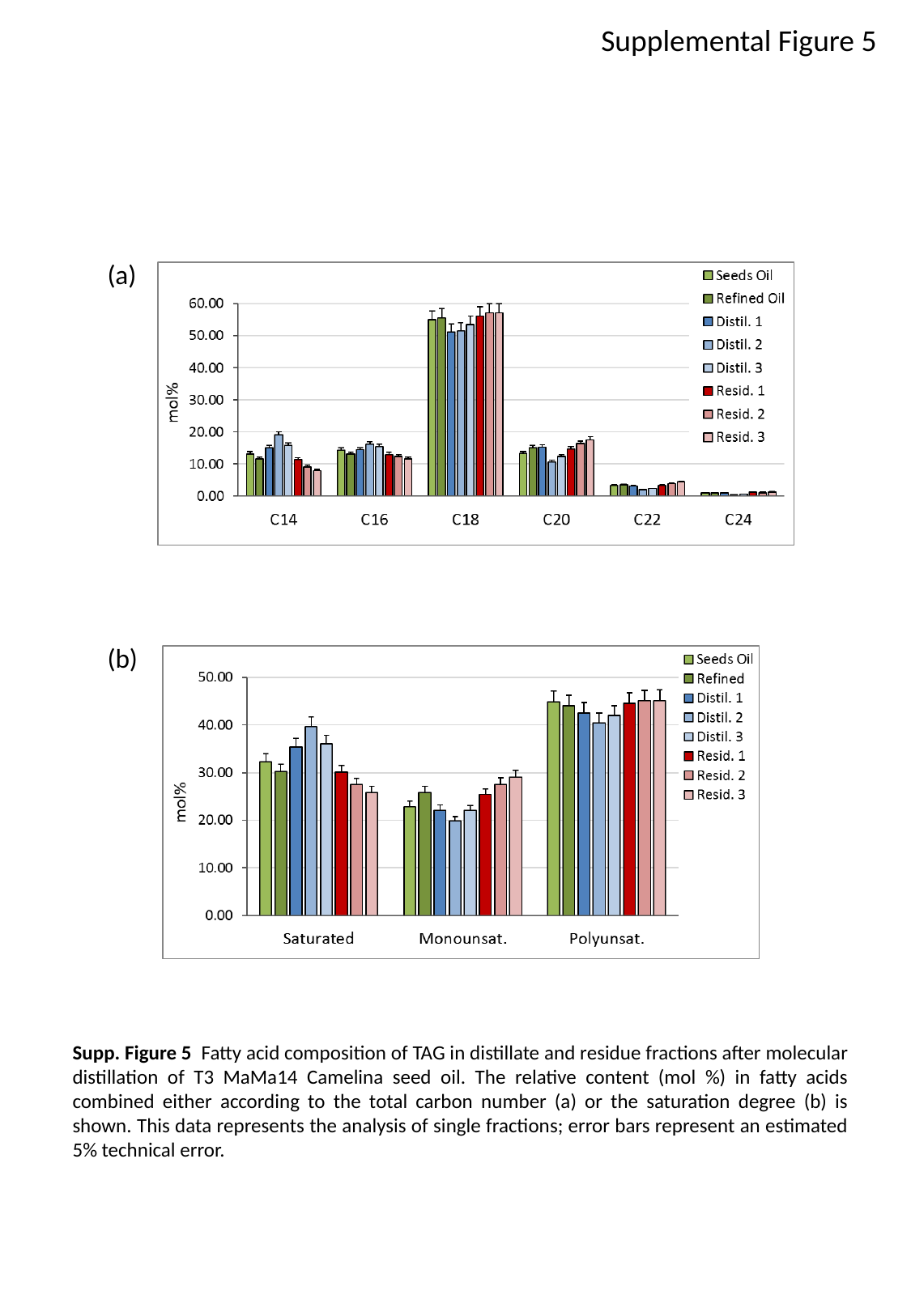

Supplemental Figure 5
(a)
(b)
Supp. Figure 5 Fatty acid composition of TAG in distillate and residue fractions after molecular distillation of T3 MaMa14 Camelina seed oil. The relative content (mol %) in fatty acids combined either according to the total carbon number (a) or the saturation degree (b) is shown. This data represents the analysis of single fractions; error bars represent an estimated 5% technical error.

## Slide 6
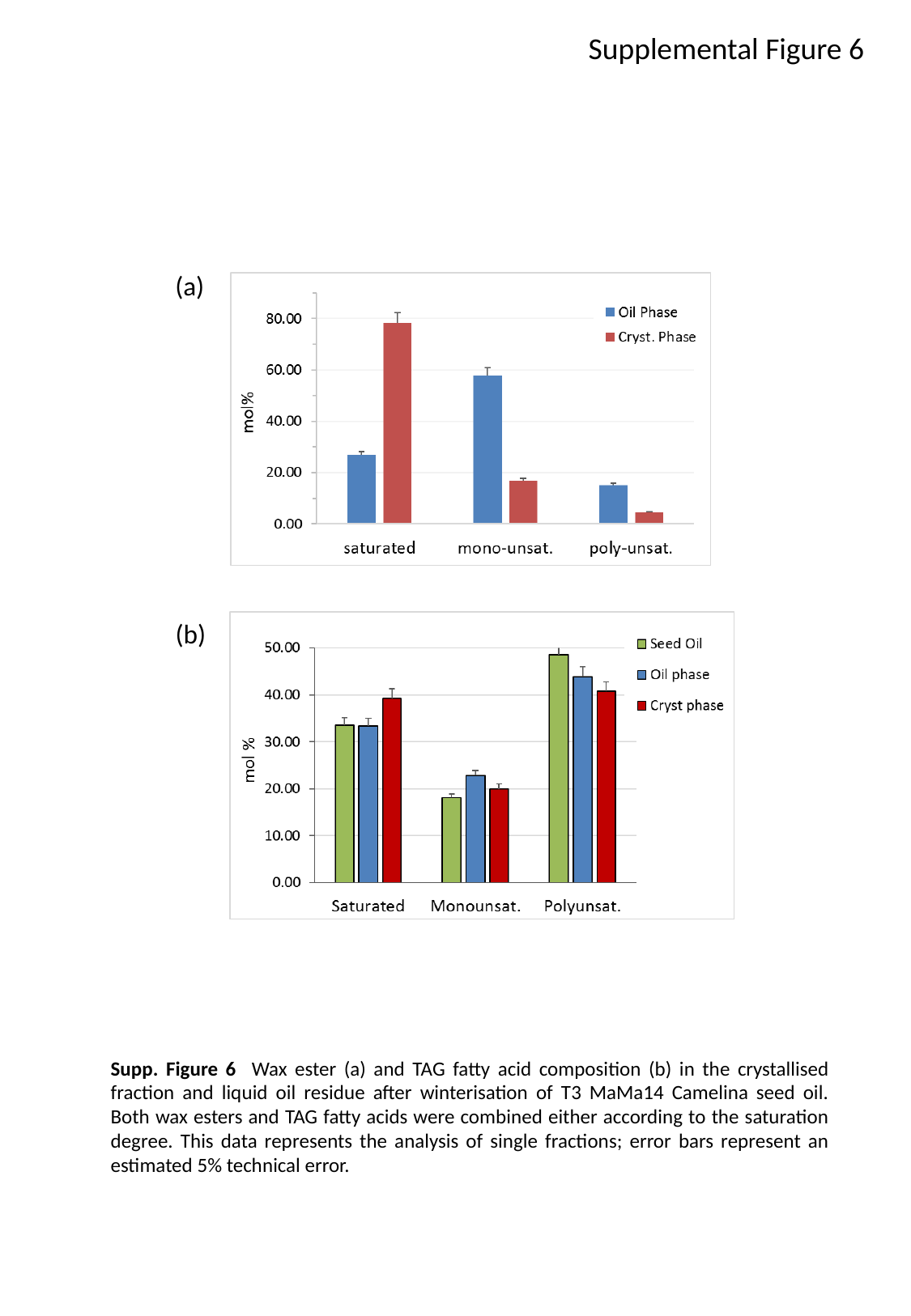

Supplemental Figure 6
(a)
(b)
Supp. Figure 6 Wax ester (a) and TAG fatty acid composition (b) in the crystallised fraction and liquid oil residue after winterisation of T3 MaMa14 Camelina seed oil. Both wax esters and TAG fatty acids were combined either according to the saturation degree. This data represents the analysis of single fractions; error bars represent an estimated 5% technical error.
