# Supplementary material for: Tailoring the composition of novel wax esters in the seeds of transgenic Camelina sativa through systematic metabolic engineering
Source: Plant Biotechnol J. 2017 Feb 2;15(7):837–49. doi: 10.1111/pbi.12679 (PMC5466440; doi:10.1111/pbi.12679)
Supplement: Supplementary file 2 — Table S1 Acyl‐CoA profiling of developing Camelina Seeds Table S2 Molecular distillation results Table S3 Oil winterisation results Table S4a Physico‐chemical properties of saturated wax esters Table S4b Physico‐chemical properties of unsaturated wax esters [file PBI-15-837-s003.docx]

**Supplementary Table 1** Acyl-CoA profiling of developing Camelina Seeds

| **Acyl CoA (%)** |  | **WT** |  | **MaMa10** | **MaMa12** | **MaMa14** |  | **MoMa10** | **MoMa12** | **MoMa14** |
| --- | --- | --- | --- | --- | --- | --- | --- | --- | --- | --- |
| **10:0** |  | 0.0 |  | 1.1 | 0.1 | 0.1 |  | 16.8 | 0.1 | 6.5 |
| **12:0** |  | 0.1 |  | 0.2 | 0.1 | 0.1 |  | 1.6 | 2.2 | 0.7 |
| **14:0** |  | 0.4 |  | 0.6 | 0.4 | 1.1 |  | 1.3 | 0.8 | 1.4 |
| **16:0** |  | 8.3 |  | 9.3 | 7.2 | 6.6 |  | 6.9 | 7.2 | 9.0 |
| **16:1** |  | 0.4 |  | 0.4 | 0.3 | 0.5 |  | 0.3 | 0.3 | 0.3 |
| **16:3** |  | 0.1 |  | 0.2 | 0.1 | 0.1 |  | 0.4 | 0.2 | 0.1 |
| **18:0** |  | 5.2 |  | 5.5 | 4.9 | 3.7 |  | 5.9 | 4.2 | 3.9 |
| **18:1** |  | 10.9 |  | 17.1 | 9.7 | 7.2 |  | 7.0 | 9.8 | 6.8 |
| **18:2** |  | 4.2 |  | 6.8 | 4.6 | 7.6 |  | 8.5 | 5.3 | 9.7 |
| **18:3** |  | 7.4 |  | 9.4 | 10.1 | 8.5 |  | 8.3 | 8.4 | 8.4 |
| **20:0** |  | 5.0 |  | 5.0 | 5.7 | 7.0 |  | 6.8 | 6.3 | 8.7 |
| **20:1** |  | 19.5 |  | 17.3 | 21.5 | 20.6 |  | 9.0 | 18.6 | 10.7 |
| **20:2** |  | 1.4 |  | 0.9 | 1.4 | 0.8 |  | 0.7 | 1.1 | 1.1 |
| **20:3** |  | 1.0 |  | 0.8 | 1.0 | 0.7 |  | 0.4 | 0.7 | 0.7 |
| **22:0** |  | 5.6 |  | 5.1 | 4.6 | 6.1 |  | 4.4 | 4.9 | 6.9 |
| **22:1** |  | 10.0 |  | 7.6 | 11.6 | 16.0 |  | 8.4 | 15.1 | 11.6 |
| **24:0** |  | 8.5 |  | 5.0 | 7.3 | 5.2 |  | 6.0 | 7.3 | 6.5 |
| **24:1** |  | 9.5 |  | 6.8 | 7.9 | 6.5 |  | 5.4 | 6.4 | 5.4 |
| **26:0** |  | 0.9 |  | 0.3 | 0.5 | 0.6 |  | 0.7 | 0.6 | 0.8 |
| **26:1** |  | 0.7 |  | 0.4 | 0.6 | 0.4 |  | 0.5 | 0.4 | 0.5 |
| **28:0** |  | 0.2 |  | 0.1 | 0.1 | 0.2 |  | 0.3 | 0.1 | 0.1 |
| **28:1** |  | 0.6 |  | 0.2 | 0.3 | 0.7 |  | 0.5 | 0.3 | 0.4 |

**Supplementary Table 2.** Molecular distillation results

| Sample | Fraction  Weight (g) | WE mg/g | WE content (g) | enrichment | % of WE  recovered |
| --- | --- | --- | --- | --- | --- |
| crude oil | 1985 | 25.04 | 49.70 |  |  |
| Distillate 1 | 19 | 120.91 | 2.30 | 4.83 | 5.18 |
| Residue 1 | 1823 | 23.08 | 42.07 | 0.92 | 94.82 |
| Refined oil | 138 | 24.65 |  |  |  |
| Distillate 2 | 27 | 54.72 | 1.48 | 2.22 | 75.34 |
| Residue 2 | 63 | 7.68 | 0.48 | 0.31 | 24.66 |
| Distillate 3 | 18 | 46.69 | 0.84 | 1.90 | 83.12 |
| Residue 3 | 21 | 8.13 | 0.17 | 0.33 | 16.88 |

**Supplementary Table 3.** Oil winterisation results

|  | **Oil weight** **(g)** | **WE mg/g oil** | **WE (g)** | **enrichment** | **% Total WE** |
| --- | --- | --- | --- | --- | --- |
| **Refined oil** | 1215 | 24.65 | 29.95 |  |  |
| **Oil Phase** | 885 | 26.74 | 23.67 | 1.08 | 79.03 |
| **Crystalised Phase** | 91 | 62.22 | 5.66 | 2.52 | 18.91 |
| **Loss** | 239 |  | 0.62 |  | 2.07 |

**Supplemental Table 4a.** Physico-chemical properties of saturated wax esters

|  |  |  | **760 mmHg** | |  | **0.1 mmHg** |  | **0.01 mmHg** |
| --- | --- | --- | --- | --- | --- | --- | --- | --- |
| **Saturated** | **WE** | **MW** | **MP**(°C) | **BP***(°C) |  | **BP****(°C) |  | **BP****(°C) |
| **C28:0** | 14:0/14:0 | 424.7 | 44.9 | 462 | ± 13 | 192 |  | 163 |
| **C30:0** | 16:0/14:0 | 452.8 | 52.0 | 485 | ± 13 | 208 |  | 177 |
| **C30:0** | 14:0/16:0 | 452.8 | 49.0 | 485 | ± 13 |  |  |  |
| **C32:0** | 16:0/16:0 | 480.9 | 54.0 | 507 | ± 18 | 223 |  | 191 |
| **C34:0** | 18:0/16:0 | 508.9 | 59.8 | 528 | ± 18 | 238 |  | 205 |
| **C34:0** | 16:0/18:0 | 508.9 | 58.7 | 528 | ± 18 |  |  |  |
| **C36:0** | 20:0/16:0 | 537.0 | 60.8 | 549 | ± 18 | 252 |  | 218 |
| **C36:0** | 18:0/18:0 | 537.0 | 61.8 | 549 | ± 18 |  |  |  |
| **C36:0** | 16:0/20:0 | 537.0 | 61.2 | 549 | ± 18 |  |  |  |
| **C38:0** | 20:0/18:0 | 565.0 | 67.6 | 569 | ± 18 | 266 |  | 231 |
| **C38:0** | 18:0/20:0 | 565.0 | 66.0 | 569 | ± 18 |  |  |  |
| **C38:0** | 16:0/22:0 | 565.0 | nd | 569 | ± 18 |  |  |  |
| **C40:0** | 20:0/20:0 | 593.1 | 69.0 | 589 | ± 18 | 280 |  | 244 |
| **C40:0** | 18:0/22:0 | 593.1 | nd | 589 | ± 18 |  |  |  |
| **C42:0** | 22:0/20:0 | 621.1 | nd | 608 | ± 23 | 293 |  | 256 |
| **C42:0** | 20:0/22:0 | 621.1 | nd | 608 | ± 23 |  |  |  |
| **C44:0** | 22:0/22:0 | 649.2 | nd | 627 | ± 23 | 306 |  | 268 |
| **C46:0** | 22:0/24:0 | 677.2 | nd | 645 | ± 23 | 318 |  | 280 |
| **C48:0** | 24:0/24:0 | 705.2 | nd | 663 | ± 23 | 331 |  | 291 |

*Values from chemical databases available at <http://www.chemspider.comand> <http://www.thegoodscentscompany.com/search2.html>

**Values estimated using Using Sigma-Aldrich's Pressure-Temperature Nomograph available at <http://www.sigmaaldrich.com/chemistry/solvents/learning-center/nomo-assets.html>

**Supplemental Table 4b.** Physico-chemical properties of unsaturated wax esters

|  |  |  | **760 mmHg** | |  | **0.1 mmHg** |  | **0.01 mmHg** |
| --- | --- | --- | --- | --- | --- | --- | --- | --- |
| **unsaturated** | **WE** | **MW** | **MP(°C)** | **BP*(°C)** |  | **BP**(°C)** |  | **BP**(°C)** |
| **C32:1** | 16:0/16:1 | 478.8 | 10.8 | 544 | ± 29 | 249 |  | 215 |
| **C32:1** | 14:0/18:1 | 478.8 | 8.3 | 544 | ± 29 | 249 |  |  |
| **C32:2** | 14:0/18:2 | 476.8 | nd | 545 | ± 29 | 250 |  |  |
| **C32:2** | 16:1:16:1 | 476.0 | -17.0 | nd |  |  |  |  |
| **C34:1** | 18:0/16:1 | 506.9 | 18.1 | 567 | ± 29 | 265 |  |  |
| **C34:1** | 16:0/18:1 | 506.9 | 17.0 | 567 | ± 29 | 265 |  |  |
| **C34:1** | 18:1/16:0 | 506.9 | 28.0 | 574 | ± 29 | 269 |  |  |
| **C36:1** | 20:0/16:1 | 534.9 | 28.7 | 589 | ± 29 | 280 |  |  |
| **C36:1** | 18:1/18:0 | 534.9 | 34.0 | 596 | ± 29 | 285 |  |  |
| **C36:1** | 18:0/18:1 | 534.9 | 24.0 | 589 | ± 29 | 280 |  |  |
| **C36:2** | 18:2/18:0 | 532.9 | nd | 597 | ± 29 | 285 |  |  |
| **C36:2** | 18:1/18:1 | 532.9 | -4.0 | 597 | ± 39 | 285 |  |  |
| **C36:2** | 18:0/18:2 | 532.9 | nd | 591 | ± 29 | 281 |  |  |
| **C38:1** | 22:0/16:1 | 563.0 | nd | 611 | ± 34 | 295 |  |  |
| **C38:1** | 20:0/18:1 | 563.0 | 30.5 | 611 | ± 34 | 295 |  |  |
| **C38:1** | 18:0/20:1 | 563.0 | nd | 612 | ± 24 | 296 |  |  |
| **C38:1** | 18:1/20:0 | 563.0 | 39.8 | 618 | ± 34 | 300 |  |  |
| **C38:2** | 20:0/18:2 | 561.0 | nd | 613 | ± 34 | 296 |  |  |
| **C38:2** | 18:2/20:0 | 561.0 | nd | 618 | ± 34 | 300 |  |  |
| **C40:1** | 22:0/18:1 | 591.0 | nd | 632 | ± 34 | 309 |  |  |
| **C40:1** | 18:1/22:0 | 591.0 | nd | 639 | ± 34 | 314 |  |  |
| **C40:2** | 22:0/18:2 | 589.0 | nd | 634 | ± 34 | 310 |  |  |
| **C40:2** | 18:2/20:0 | 589.0 | nd | 639 | ± 34 | 314 |  |  |
| **C42:1** | 24:0/18:1 | 619.0 | nd | 653 | ± 34 | 324 |  | 285 |
| **C44:1** | 24:1/20:0 | 647.1 | nd | 669 | ± 24 | 335 |  | 295 |
| **C44:1** | 24:0/20:1 | 647.1 | nd | 669 | ± 24 | 335 |  | 295 |
| **C44:1** | 20:1/24:0 | 647.1 | nd | 669 | ± 24 | 335 |  | 295 |
| **C44:1** | 20:0/24:1 | 647.1 | nd | 669 | ± 24 | 335 |  | 295 |
| **C44:2** | 20:1/24:1 | 645.1 | nd | 668 | ± 24 | 334 |  | 295 |
| **C44:2** | 24:1/20:1 | 645.1 | nd | 668 | ± 24 | 334 |  | 295 |
| **C46:2** | 24:1/22:1 | 673.2 | nd | 688 | ± 24 | 348 |  | 307 |
| **C46:2** | 22:1/24:1 | 673.2 | nd | 688 | ± 24 | 348 |  | 307 |
