# Supplementary material for: Tailoring the composition of novel wax esters in the seeds of transgenic Camelina sativa through systematic metabolic engineering
Source: Plant Biotechnol J. 2017 Feb 2;15(7):837–49. doi: 10.1111/pbi.12679 (PMC5466440; doi:10.1111/pbi.12679)
Supplement: Supplementary file 3 — Supplementary Legends [file PBI-15-837-s001.docx]

**Molecular distillation of crude and refined seed oil.**

We initially attempted to distillate WE from crude oil (produced by pressing, filtering and solvent extraction) for 2.5 h at 240 °C and under limited vacuum (from 0.002 to 1.6 mbar) (Fig. 7a). The resulting distillate fraction was enriched 4.8 time in WE but contained only 5% of the total WE present in the oil (supp. Table 2; Distillate 1). Analysis of the WE content in this fraction revealed that it was enriched in short chain (C28-C34) saturated molecular species (Supp. Fig. 2a) but, interestingly, depleted of polyunsaturated species (Supp. Fig. 2b). This illustrates the fact that desaturation of acyl chains results in lower melting point of hydrocarbon compounds but inversely in higher boiling point (Supp. Table 4a and b) and hence is consistent with the less efficient extraction of unsaturated WEs observed in these experimental conditions. These results indicated that both temperature and vacuum were not elevated enough to recover longer (C36 and over) and polyunsaturated WEs and yet both the distillate and residue fractions were very dark (Fig. 7b; R1 and D1) indicating that refining of the oil was required before elevating distillation temperature. These results also indicated the need for a more powerful vacuum to be applied to the system to prevent burning and to lower the boiling point of longer WEs. A fraction of residue 1 was refined following standard a procedure (hydration, degumming and neutralisation) used in the vegetable oil processing industry (Supp. Figure 2a). This yielded a paler oil cleared of free fatty acids, phospholipids and lipid oxidation products (Fig. 7b; RO). An aliquot of this refined oil was subjected to MD at 300 °C for 1.5 h under stronger vacu*um* (0.003 to 0.12 mbar) and this time quantification by HPLC revealed that over 75% of the WEs recovered were in the distillate fraction (Supp. Table 2; Distillate 2). GC analysis of WEs in all fraction confirmed that residue 2 was largely depleted of WEs (Supp. Figure 3) and comparison with crude seed oil composition showed that WEs were not degraded and all efficiently exacted (Supp. Figure 4). This was confirmed by qualitative analysis which demonstrated that distillate 2 had a similar WE composition to that of both the initial residue 1 fraction and the refined oil aliquot used (Supp. Fig 2a). However, only a moderate 2.2-fold enrichment was achieved (Supp. Table 2) due to co-distillation of TAG species. Analysis of the fatty acid composition of TAGs in the distilled fractions also showed a slight bias toward shorter (C14 and C16) saturated acyl chains (supp. Figure 5) consistent with WE profiles in distillate 1 (Supp. Figure 2). A third distillation attempt, achieving significantly stronger vacuum at terminal temperature (Fig. 7a), improved WE extraction with 83% of WE in Distillate 3 but also resulted in more TAG co-distillation and hence a slightly less efficient enrichment (1.9-fold; Supp. Table 2).

**Oil winterisation**

1215 g of the refined oil described above was gradually cooled down to 4°C and a 91g crystallised fraction was separated from liquid oil by filtration (Supp. Table 3). Analysis of this fraction by HPLC revealed a modest 2.5-fold enrichment in WEs compared to refined oil (62.22 and 24.65 mg WE / ml oil respectively; Supp. Table 3) suggesting that some TAG species also solidified in these experimental conditions. In addition, only 18.9 % of the total WE could be recovered using this method the vast majority remaining in the liquid oil phase. This result was surprising because the WE produced by the MaMa14 line used in this experiment contain over 40% saturated species and the melting point of the shortest compound (C28:0) is well above winterisation temperature (45 °C and 4 °C respectively). Analysis of the WEs composition in both the crystallised and liquid oil fractions confirmed that the solid fraction recovered very dramatically enriched in saturated WEs (80% of total WEs in That fraction; Supp Figure 6a). GC analysis of the fatty acid content of TAG present in the crystallised fraction only showed a slight enrichment in saturated acyl chains compared to the liquid oil phase confirming that the majority of TAG molecular species solidified and partitioned with the crystallised esters (Supp. Figure 6b).
